# Species identification based on a semi-diagnostic marker: Evaluation of a simple conchological test for distinguishing blue mussels *Mytilus edulis* L. and *M*. *trossulus* Gould

**DOI:** 10.1371/journal.pone.0249587

**Published:** 2021-07-23

**Authors:** Vadim Khaitov, Julia Marchenko, Marina Katolikova, Risto Väinölä, Sarah E. Kingston, David B. Carlon, Michael Gantsevich, Petr Strelkov

**Affiliations:** 1 St. Petersburg State University, St. Petersburg, Russia; 2 Kandalaksha State Nature Reserve, Kandalaksha, Murmansk Region, Russia; 3 Murmansk Marine Biological Institute, Murmansk, Russia; 4 Finnish Museum of Natural History, University of Helsinki, Helsinki, Finland; 5 Department of Biology & Schiller Coastal Studies Center, Bowdoin College, Brunswick, Maine, United States of America; 6 School of Marine Sciences and Darling Marine Center, University of Maine, Walpole, Maine, United States of America; 7 Department of Invertebrate Zoology, Lomonosov Moscow State University, Moscow, Russia; 8 Laboratory of Monitoring and Conservation of Natural Arctic Ecosystems, Murmansk Arctic State University, Murmansk, Russia; Helmholtz-Zentrum fur Ozeanforschung Kiel, GERMANY

## Abstract

Cryptic and hybridizing species may lack diagnostic taxonomic characters leaving researchers with semi-diagnostic ones. Identification based on such characters is probabilistic, the probability of correct identification depending on the species composition in a mixed population. Here we test the possibilities of applying a semi-diagnostic conchological character for distinguishing two cryptic species of blue mussels, *Mytilus edulis* and *M*. *trossulus*. These ecologically, stratigraphically and economically important molluscs co-occur and hybridize in many areas of the North Atlantic and the neighboring Arctic. Any cues for distinguishing them in sympatry without genotyping would save much research effort. Recently these species have been shown to statistically differ in the White Sea, where a simple character of the shell was used to distinguish two mussel morphotypes. In this paper, we analyzed the associations between morphotypes and species-specific genotypes based on an abundant material from the waters of the Kola Peninsula (White Sea, Barents Sea) and a more limited material from Norway, the Baltic Sea, Scotland and the Gulf of Maine. The performance of the “morphotype test” for species identification was formally evaluated using approaches from evidence-based medicine. Interspecific differences in the morphotype frequencies were ubiquitous and unidirectional, but their scale varied geographically (from 75% in the White Sea to 15% in the Baltic Sea). In addition, salinity-related variation of this character within *M*. *edulis* was revealed in the Arctic Barents Sea. For every studied region, we established relationships between the proportions of the morphotypes in the populations as well as between the proportions of the morphotypes in samples and the probabilities of mussels of different morphotypes being *M*. *trossulus* and *M*. *edulis*. We provide recommendations for the application of the morphotype test to mussels from unstudied contact zones and note that they may apply equally well to other taxa identified by semi-diagnostic traits.

## Introduction

Blue mussels *Mytilus edulis* and *M*. *trossulus* are old evolutionary lineages of Pliocene origin [1]. The more common *M*. *edulis* is thought to be native in the Atlantic, while the originally Pacific *M*. *trossulus* has colonized the northwest Atlantic in a series of natural and anthropogenic invasions [2–4]. Now these two species co-occur and hybridize in at least six geographical sectors of the North Atlantic and the adjacent Arctic coasts: western Greenland, American coast from the Gulf of Maine to Hudson Bay, northeastern Scotland, western Baltic Sea, western Norway and the coasts of the Kola Peninsula in Russia (White Sea, Barents Sea) ([5] and references therein).

Ever since the existence of *M*. *trossulus* was recognized by molecular genetic markers (allozymes) [6], the search has been on for reliable morphometric characters allowing one to distinguish it from *M*. *edulis*. The discreteness of these two species was confirmed in studies employing numerous metric shell traits in a multivariate approach, but no individually diagnostic characters have been found [7–10]. Therefore *M*. *edulis* and *M*. *trossulus* are generally treated as cryptic species and are routinely identified by molecular markers. While multilocus analysis is desirable for an unambiguous identification of species and their hybrids, in practice singular presumably diagnostic markers are usually employed, most often the coding region of the polyphenolic adhesive protein gene (ME 15/16 or Glu-5’) [11].

*Mytilus edulis* and *M*. *trossulus* are ecologically, economically and stratigraphically important molluscs [12–14]. Apart from their different biogeographic histories, these two species are known or suspected to differ in life traits, ecological requirements and properties as biomonitoring and aquaculture objects [15–18]. An illustrative example is the harm associated with *M*. *trossulus* invasion on longline aquaculture designed for *M*. *edulis*. A cryptic presence of *M*. *trossulus* in *M*. *edulis* plantations in Loch Etive (Scotland) in the 2000s resulted in significant production losses because *M*. *trossulus* had lower consumer properties and shells too fragile for harvesting and grading [19, 20]. Considerable differences between species were also found in Canadian aquaculture [8, 21], where the commercial value of *M*. *trossulus* was estimated to be 1.7 times less than that of *M*. *edulis* [8]. The difficulty of identifying *M*. *edulis* and *M*. *trossulus* by the shells is frustrating, and any cue for distinguishing these species in sympatry without genotyping would be a welcome addition to the toolkit of mussel studies.

We have recently discovered that *M*. *edulis* and *M*. *trossulus* in the White Sea differ by a simple conchological trait: the presence or absence of an uninterrupted prismatic strip under the ligament on the inner side of the shell. This strip is found in 74% of *M*. *trossulus*-like mussels (i.e. mussels with multilocus genotypes dominated by *M*. *trossulus* genes; this group mostly consists of purebreds but also includes some hybrids), while 96% of *M*. *edulis*-like mussels lack this character [16, 22]. Hence we denote the mussels that bear the strip as the T-morphotype and those that lack this strip, as the E-morphotype.

This finding raises two questions. The first is how to apply this marker for individual and population assignment correctly and efficiently. Species identification is usually based on fixed diagnostic traits, which have a unique state for all individuals of a species. The conchological trait under consideration is not diagnostic but semi-diagnostic, i.e. polymorphic within species but with states distributed in different frequencies across species (see [23]). Since there are strong (70%) differences in the morphotype frequencies between the mussel species in the White Sea, one can fall into a trap of deciding that any T-morphotype mussel from the White Sea can be assigned with a high probability to *M*. *trossulus* while any E-morphotype mussel can be assigned to *M*. *edulis*. In fact, however, the probabilities of correct identification depend on the proportions of *M*. *trossulus* and *M*. *edulis* in the mixed population under study. Any mussel sampled from a “pure” *M*. *trossulus* population (an expected T-morphotype frequency *PT* = 74%) would be *M*. *trossulus* regardless of the morphotype. By the same token, any mussel sampled from a “pure” *M*. *edulis* population (*PT* = 4%) would be *M*. *edulis*. At the same time, in a 1:1 mixture of species (expected *PT* = (74+4)/2 = 39%), 95% of the T-morphotypes would be *M*. *trossulus* (*P(tros|T)* = 0.74x0.5/(0.39) = 0.949), while 79% of the E-morphotypes would be *M*. *edulis* (*P(edu|E)* = 0.96x0.5/(1–0.39) = 0.787). However, these calculations are valid only if the morphotype frequencies within ‘species-specific’ genotypes do not vary with the proportions of species in mixed populations (“taxonomic structure of populations” hereinafter).

In such a situation, taxonomists may profit from the experience of clinicians. They often have to deal with semi-diagnostic characters since many clinical diagnostic tests employ semi-diagnostic markers. A formal procedure has been developed in evidence-based medicine to evaluate the ability of clinical tests to classify patients as having or not having the target condition relative to the reference standard (e.g. [24]). We suggest that this methodology might be useful for the evaluation of taxonomic tests for cryptic species relative to the species-specific genotype. To emphasize the analogy with the clinical approach, we refer to the procedure of mussel species identification based on the morphotype as the “morphotype test”.

The second question is whether the basic morphological differences between *M*. *trossulus* and *M*. *edulis* revealed in the White Sea are a local phenomenon or whether these two species can be distinguished by the morphotype in other populations and contact zones as well. Should the latter prove true, the morphotype test would facilitate local mussel studies in the Atlantic. Since interspecific differences in this particular character were overlooked in previous morphometric studies, which all were based on references from outside of the White Sea [7–10], it remains possible that this difference is indeed valid only in the White Sea. Such a situation could be associated with the unusual environment, featuring a combination a subarctic climate and a relatively low salinity (< 25 ppt—[25]) and/or with the history of the local *M*. *trossulus*. *M*. *trossulus* is thought to have invaded the Kola Peninsula with marine traffic only recently, in the middle of the 20th century, while most other Atlantic populations are probably much older [3].

In this paper we address the above two questions. Firstly we analyze the associations between morphotypes and species-specific genotypes in an abundant material from the waters of the Kola Peninsula and in more limited material from Norway, the Baltic Sea, Scotland and the Gulf of Maine. For the Kola material, we compare populations from the marginal, semi-enclosed White Sea and from the oceanic Barents Sea coasts on the one hand, and populations from the brackish vs saline localities in the Barents Sea on the other hand. The purpose is to see how local geography and salinity (or associated factors) affect morphotype frequencies in populations with similar biogeographic histories existing under similar climatic conditions. Secondly, we formally evaluate the performance of the “morphotype test” for species identification using approaches from evidence-based medicine, and provide practical recommendations for its use for population and individual assessment.

## Materials and methods

### Samples

**Fig 1:**
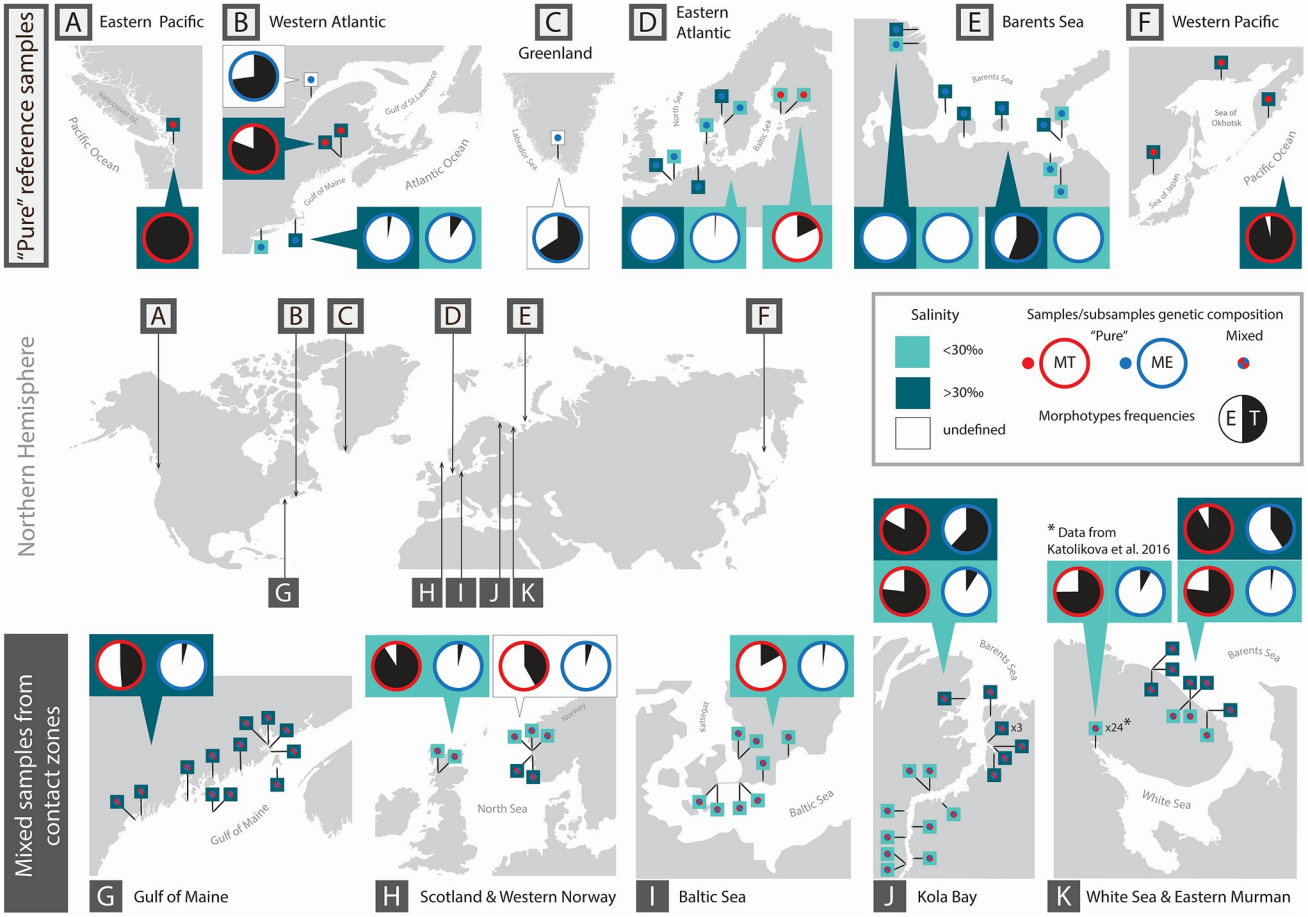
Map of the study area and variation in shell morphotype frequencies. The bottom panel (maps G-K) shows five geographical contact zones between *M*. *edulis* and *M*. *trossulus*, maps in the upper panel (A—F)—other studied areas. Pins depict sampling sites. Pie diagrams depict proportions of T-morphotypes (black sector) and E-morphotypes (white sector) in *M*. *trossulus* (diagrams with a red border) and *M*. *edulis* (those with a blue border) in combined samples from particular regions. If the data on salinity in sampling localities are available and considered in the analyses, it is indicated by the color of pins (light green–brackish, dark green–saline, white–salinity is unknown) and the proportions of the T-morphotypes in combined samples from brackish and saline localities are presented separately in diagrams placed on light and dark green background, respectively. Source data are given in S1 Table and S2 Table. Inkscape 0.92 [26] was used for producing the map.

Altogether, we considered 77 mussel samples (total sample size N = 4304, individual sample size N = 18–173) representing five geographical contact zones between *M*. *edulis* and *M*. *trossulus*: the Gulf of Maine in the northwestern Atlantic (12 samples, N = 428), Loch Etive in northern Scotland (2 populations, N = 160), western Baltic Sea (8 samples, N = 601), Bergen city area in western Norway (5 samples, N = 365) and the coasts of the Kola Peninsula in northern Russia: 24 samples from the White Sea (N = 1105) and 26 samples from the Barents Sea (N = 1645) (Fig 1). Detailed information about samples and sampling localities is provided in the S1 Table.

Samples in Gulf of Maine were permitted by Maine Department of Marine Resources (Special License Numbers ME 2014-41-00 and 2015-51-01) and Fisheries and Oceans Canada 2015 (Licence 341852). No special permits were required for the field studies carried out in other regions.

The Barents Sea samples were taken in the Kola Bay and at the open oceanic coast of the eastern Murman. Based on the salinity in the sampling localities, they were classified into brackish (salinity 5–30 ppt) and saline (>30 ppt). The first group consisted of nine samples from the freshened top of the Kola Bay and three samples from the open coast. The second group consisted of eight samples from the mouth of the Kola Bay and six samples from the open coast (Fig 1).

As for the samples from the other contact zones, all American samples and two out of five Norwegian samples were from saline habitats, while all the others were from brackish habitats. Salinity conditions in the sampling localities were either taken from the literature [27–32] or, in case of the few American and the Barents Sea open coast localities, predicted based on the presence or absence of large rivers nearby.

In addition to the samples taken in the five contact zones, we identified the morphotypes in 27 samples (total sample size N = 912, individual sample size N = 12–76) of supposedly pure blue mussel species from distant localities: *M*. *trossulus* from Passamaquoddy Bay and *M*. *edulis* from the Gulf of Saint Lawrence in eastern Canada, *M*. *trossulus* from the northern Baltic Sea, from Puget Sound in eastern Pacific and from multiple areas of western Pacific, *M*. *edulis* from southwestern Greenland, from the Long Island Sound and Cape Cod in the eastern USA, and from saline and brackish localities in Europe and in the southwestern Barents Sea (Fig 1, S2 Table). To note, the Baltic *M*. *trossulus* is more strongly introgressed by *M*. *edulis* alleles than other populations [33]. Information about the species identity of regional populations and salinity conditions in sampling localities was taken from the literature. Taxonomic affinities of mussels from Canada and from western Norway, where both species could be expected, were confirmed genetically (see S2 Table for details).

### Genetic characters

A part of the material, from the contact zones, was genotyped in previous studies [3, 16, 31]. The other samples were collected and genotyped specially for this study (see S1 Table). The Gulf of Maine mussels, both from previous and new material, were genotyped using 109 260 SNPs (single nucleotide markers) as described in [31] (including some material from [34]). The mussels from the other areas were genotyped using various sets of allozyme loci, which, as a rule, included the Est-D, Gpi, Pgm and Odh loci. These four loci were involved in the initial diagnosis of *M*. *edulis* and *M*. *trossulus* and in description of all the contact zones under consideration [6, 19, 35]. They individually show 70–95% allele frequency differences between the species [6], and, being less affected by introgression than most of the conventionally used PCR-based “diagnostic” markers [1, 36], are reliable markers for species identification everywhere. The other new samples were genotyped by Est-D, Gpi, Pgm and Odh as in [16]. For seven samples from the Barents Sea the data on only three loci (Est-D, Gpi and Pgm) were available (see S1 Table). The SNP data set and each of the four regional 4-locus allozyme sets (from the Baltic, Norway, Scotland and Russia) were analyzed separately using STRUCTURE or fastSTRUCTURE software ([37], settings as in [16] and [31]). The obtained q-values are defined as estimates of the proportion of *M*. *trossulus* genes in individual genotypes (proportion of *M*. *edulis* genes is therefore 1-q). The material from Russia was also analyzed by three loci (all but Odh) to show that the exclusion of Odh did not affect the inference. The mussels were further classified into two categories by their q-values: genotypes dominated by *M*. *trossulus* genes (q-value > 0.5) and those by *M*. *edulis* genes (q-value ≤ 0.5). For ease of presentation, these categories will be referred to as “*M*. *trossulus*” and “*M*. *edulis*” genotypes although each includes both purebreds and hybrids.

### Morphological characters

Data on the White Sea samples were taken from [16] and the other samples were processed accordingly. We measured the maximum length of each shell to the nearest 0.1 mm with electronic calipers and investigated the inner surface of the valves under a dissecting stereo-microscope. A mussel was classified as a T- or an E-morphotype based on, respectively, presence or absence of an uninterrupted strip of the prismatic layer under the ligament on the inner side of the shell.

### Predictive values

For each sample we calculated the frequencies of *M*. *trossulus* genotypes (*Ptros*) and of T-morphotypes (*PT*) and four indices reflecting the strength of association between genotypes and morphotypes: *P(T|tros)*–the proportion of T-morphotypes among *M*. *trossulus*, *P(E|edu)*–the proportion of E-morphotypes among *M*. *edulis* (for practical reasons we used *P(T|edu)* = 1- *P(E|edu)*, the proportion of T-morphotypes among *M*. *edulis*), *P(tros|T)*–the proportion of *M*. *trossulus* among T-morphotypes, *P(edu|E)*–the proportion of *M*. *edulis* among E-morphotypes. *P(tros|T)* and *P(edu|E)* are the key indices because they show, respectively, how likely it is that a randomly taken T-morphotype mussel is *M*. *trossulus* and a randomly taken E-morphotype mussel, *M*. *edulis*.

Here we would like to offer an analogy between the indices used in our study and those used in clinical medicine for evaluating the performance of diagnostic tests. If we consider *M*. *edulis* as a “healthy” mussel and *M*. *trossulus* as a “sick” mussel (which is not so far-fetching considering the threat presented by *M*. *trossulus* to the Scottish aquaculture [19]), then our terms have the following medical equivalents [24]: *Ptros* is *prevalence*, *P(T|tros)* is *sensitivity*, *P(E|edu)* is *specificity*, *P(tros|T)* is *positive predictive value* and *P(edu|E)* is *negative predictive value* of the morphotype test.

As with clinical tests where disease markers are not 100% sensitive, the positive and negative predictive values will depend on the prevalence, i.e. the frequency of the species (or disease) of interest in the material [24]. With increasing *Ptros*, *P(tros|T)* will gradually increase from 0 in pure populations of *M*. *edulis* to 1 in pure populations of *M*. *trossulus*, while *P(edu|E)* will show an opposite relationship. For the test to be meaningful, predictive values should be >0.5 since a predictive value of 0.5 indicates a random association between the genotype and the morphotype. Assuming that sensitivity and specificity do not depend on the prevalence (though this assumption may be violated, see below), predictive values could be directly predicted basing on the *Ptros* in a sample and the known sensitivity and specificity using the formulas:

$$P(tros|T)=\frac{Ptros\cdot P(T|tros)}{Ptros\cdot P(T|tros)+(1-Ptros)\cdot P(T|edu)}$$ (1)

$$P(edu|E)=\frac{\left(1-Ptros)\cdot (1-P(T|edu)\right)}{\left(1-Ptros)\cdot (1-P(T|edu))+Ptros\cdot (1-P(T|edu)\right)}$$ (2)

The prevalence (*Ptros*) in turn could be predicted based on *P(T|edu)*, *P(T|tros)* and *PT* in a sample:

$$Ptros=\frac{PT-P(T|edu)}{P(T|tros)-P(T|edu)}$$ (3)

### Statistical analyses

After the examination of the associations between morphotypes and individual q-values within sample sets representing different contact zones, the following analyses were made. Firstly, we studied variation of *PT*, *P(T|tros)*, *P(T|edu)*, *P(tros|T)*, *P(edu|E)* as functions of *Ptros* within and between sample sets representing A) the White Sea (sample set *WS*) and the Barents sea coasts of the Kola Peninsula and saline (set *BH*) and brackish (set *BL*) water localities in the Barents Sea (Section “Associations between morphotypes and genotypes around the Kola Peninsula”), B) different geographical contact zones between species. Whenever possible, formulas describing empirical relationships between *Ptros* and *PT* and between positive (*P(tros|T)*) and negative (*P(edu|E)*) predictive values and *Ptros* were derived on the basis of regression analysis (Section “Associations between morphotypes and genotypes around the Atlantic”). Secondly, we analyzed genotype-specific associations between morphotypes and the shell size in order to verify the hypothesis that morphological variation under consideration is not related to mussel size (Section “Associations between morphotypes and shell size”). Finally, we tested how well *Ptros*, *P(edu|E)* and *P(tros|T)* could be predicted using formulas Eqs 1–3 and the data on the morphotype proportions among species (*P(T|tros)*, *P(T|edu)*) in a few (minimum two, see below) genotyped samples. We concede that the assumption that sensitivity and specificity do not depend on the prevalence can be violated in the morphotype test, as it often is in clinical tests [38]. Therefore we focused on finding out which samples were better suited for prediction on the basis of Eqs 1–3: the most mixed samples (*Ptros*~0.5) or the combination of the two most pure samples of each species. The samples identified in this way as best suited for prediction can be used as “calibrating” ones (Section “Prediction of taxonomic structure of populations and predictive values of the morphotype test based on probability calculators”).

All statistical analyses were performed with functions of R3.6.1 statistical programming language [R_2019]. We used generalized linear (mixed) models, GL(M)Ms, with binomial distribution and a logit link-function. All GLM models were constructed with glm() function from the package “stats” [R_2019] whereas GLMM were fitted with glmer() function from the package “lme4” [39]. The validity of each model was checked by visual analysis of residual plots and the assessment of the overdispersion presence.

The goodness of fit for the models was assessed by means of pseudo-R^2^ [40] using the function r.squaredGLMM() from the package “MuMIn” [41]. To assess the role of random factors in GLMM, we compared marginal and conditional pseudoR^2^ [40]. After the model parameters were estimated, we visualized them by means of regression lines with corresponding 95% confidence intervals.

*Associations between morphotypes and genotypes around the Kola Peninsula*. The following three regression models were fitted for the data:

**Model 1**: Morphotype proportions (*PT*) as a function of taxonomic structure of mussel populations (*Ptros*). All mussels with a T-morphotype were coded as 1 and all mussels with an E-morphotype were coded as 0. These data were used as a dependent variable, which was regressed against *Ptros* (continuous predictor) and **Set** (discrete predictor with three levels) and interaction between them.
**Model 2**: Morphotype proportions among species (*P(T|tros)*, *P(T|edu)*) as a function of taxonomic structure of populations (*Ptros*). The dependent variable was coded as in Model 1 and modeled as a function of *Ptros*, *Set*, *Species* (a discrete predictor with two levels) and interaction between terms. The sample was included into the model as a random factor influencing the model intercept.
**Model 3**: Correctness of species identification (*P(tros|T)* and *P(edu|E)*) as a function of taxonomic structure of populations. The dependent variable was coded as 1 if *M*. *trossulus* was represented by a T-morphotype or *M*. *edulis* was represented by an E-morphotype and as 0 in the other cases. The set of predictors for the model was as follows: *Ptros*, *Morphotype* (discrete predictor with two levels), *Set* and interaction between terms. The sample was included into the model as a random factor influencing the model intercept.
  To check whether it is possible to pool some of the geographical sets to construct a more general model without losing information, we constructed three complex data sets with different pairing combinations of *WS*, *BL* and *BH*: (*WSBL*) and *BH*; (*WSBH*) and *BL*; (*BLBH*) and *WS*. We did not consider a full combination of sets since in such a case the factor “*Set*” would be discarded from the model. We applied the structure of Model 3 to these new recombined datasets. Then we compared AICs of these new models with AIC of Model 3 based on non-pooled data. If AIC of a new model was less than the AIC of the initial one, we considered this as a basis for pooling of the corresponding sample sets.
  *Associations between morphotypes and genotypes around the Atlantic*. Five sample sets were considered, representing the Gulf of Maine (*GOM*), the Baltic Sea (*BALT*), western Norway (*NORW*), saline Barents Sea (*BH*) and the White Sea combined with the brackish Barents Sea (*WSBL*, sets *WS* and *BL* were pooled since there pooling did not affect the inference, see Results). Scotland (*SCOT*) was not included in regression analyses because it was represented by only two samples. Three models were constructed:
**Model 4**. Taxonomic structure (*Ptros*) as a function of morphotype frequencies in populations (*PT*). The dependent variable was coded as in Model 1 and modeled as a function of *PT* (continuous predictor), *Set* and interaction between them. We modeled *Ptros* vs. *PT* but not vice versa, as in the previous analysis, in order to use this model as a reference for the “*Ptros* by *PT* calculator” (see below).
**Model 5**. Morphotype proportions among species (*P(T|tros)*, *P(T|edu)*) as a function of taxonomic structure of populations (*Ptros*). The model was constructed analogously to Model 2.
**Model 6**. Correctness of species identification (*P(tros|T)* and *P(edu|E)*) as a function of taxonomic structure of populations (*Ptros*). The model was constructed analogously to Model 3.

*Associations between morphotypes and shell size*. To check the possible association of morphotypes with size we undertook the following two analyses. Firstly, we constructed a set logistic regression models for each available species-specific genotype (i.e. *M*. *edulis* or *M*. *trossulus*) from each sample. The probability of the presence of the T-morphotype was a dependent variable and mussel size was a predictor in these models. Only cases where slope-terms of the models were statistically significant (p < 0.05) after Hochberg’s correction for multiple testing [42] were considered. Secondly, we checked the presence of any patterns in residuals from Model 6 (i.e. the main model designed to predict the probability of correct identification of an individual mussel by its morphotype) as a function of mussel size.

#### Prediction of taxonomic structure of populations and predictive values of the morphotype test based on probability calculators

We applied Eqs 1–3 to predict *Ptros*, *P(edu|E)* and *P(tros|T)* for samples from each data set (*GOM*, *BALT*, *NORW*, *BH*, *WSBL*, *SCOT*) using estimates of morphotype proportions among species (*P(T|tros)*, *P(T|edu)*) obtained from combinations of “calibrating” samples selected based on the results of the following analysis.

We considered all 630 possible pairwise combinations of samples from the *WSBL* dataset. Each pair was characterized by an index of taxonomic similarity between the samples:

$$Delta=\left(Ptros1\right)*\left(1-Ptros2\right)+\left(Ptros2\right)*\left(1-Ptros1\right),$$ (4)

where *Ptros*1 and *Ptros*2 –higher and lower estimates of prevalence in samples. The index varies in a range [0; 1] and takes the value Delta = 0 when both samples are pure *M*. *edulis* (*Ptros*1 = *Ptros*2 = 0) or pure *M*. *trossulus* (*Ptros*1 = *Ptros*2 = 1), Delta = 0.5 when both samples are equivalent mixtures of two species (*Ptros*1 = *Ptros*2 = 0.5) and Delta = 1 when one sample represent pure *M*. *trossulus* (*Ptros*1 = 1) and another pure *M*. *edulis* (*Ptros*2 = 0).

Estimates of *P(T|tros)*, *P(T|edu)* and *PT* were obtained from pooled data on each pair of samples and used for calculation of predicted values of *P(edu|E)* and *P(tros|T)* basing on Eqs 1 and 2 for the range of *Ptros* [0;1] with the step 0.01 (“genotype by morphotype calculator”) and predicted values of *Ptros* basing on Eq 3 for the range of *PT* [0;1] with the step 0.01 (“*Ptros* by *PT* calculator”). (Note that dealing with Eqs 1 and 2 we assume that *Ptros* is known while in reality it should be assessed using Eq 3). Values of *P(edu|E)* and *P(tros|T)* obtained by Eqs 1 and 2 were contrasted the ones predicted by the Model 6, and values of *Ptros* obtained by Eq 3 were compared with predictions of Model 4 using correspondence statistics:

Goodness=1/MSS, (5)

where MSS–mean sum of squares of difference between predictions of regression model and predictions of equation. Goodness varies (0; infinity) and approaches zero when predictions of models agrees poorly.

Goodness indices for each pair were plotted against the corresponding Delta values and the LOESS regression curve was fitted to find associations between them. Depending on the results of the analyses, we determined which combinations of samples could be used for predictions with best results. Best combinations of samples from each set were used to assess *P(T|edu)* and *P(T|tros)* as parameters of Eqs 1–3. Then we calculated predictions from Eq 3 for the range of *PT* and predictions from Eqs 1 and 2 for the range of *Ptros*. These predictions were visually compared with those from regression Model 4 and Model 6, respectively.

Additionally, we tested the “lazy *Ptros* by *PT* calculator” which assumes that samples with the highest and the lowest *PT* in regional sample sets do represent, respectively, pure *M*. *trossulus* and pure *M*. *edulis* and that morphotype frequencies in these samples could be directly used as parameters *P(T|tros)* and *P(T|edu)* of Eq 3. *Ptros* values predicted by the calculator for samples from different sets were visually compared with empirical ones.

For illustrative purposes and for the convenience of potential users of the “morphotype test” or any similar semi-diagnostic tests we provide the online “*Ptros* by *PT*” and “genotype by morphotype” probability calculators implementing Eqs 1–3 at https://polydora.shinyapps.io/Calculator/.

## Results

### Geographical variation in mussel morphotypes

The studied binary morphological character was previously defined as the “presence/absence of a distinct uninterrupted dark prismatic strip under the ligament”, based on material from the White Sea only [16, 22]. In the broader material of this study, encompassing different geographical zones, the E-morphotypes looked the same everywhere and conformed to the previous description: the strip was absent, and the nacreous layer totally or partially covered the space under the ligament nympha (S1A and S1C Fig). However, T-morphotypes showed some variation previously unrecorded in the White Sea. Firstly, most of the populations contained, though rarely, shells in which the nacreous-free strip of the prismatic layer was quite narrow and looked like a stria rather than a strip. Secondly, in all T-morphotypes from the Gulf of Maine and in rare T-morphotypes from the other populations the strip was not dark but pale, as the prismatic layer itself. Such T-morphotypes were difficult to notice by an unaided eye, but could be unambiguously identified with a dissecting microscope, by the presence of a pronounced scar defining the boundary of the nacreous layer under the ligament nympha (S1 Fig).

Therefore, we amend the description of the T/E morphotype character as follows: the presence/absence of an uninterrupted strip of the prismatic layer under the ligament nympha clearly recognizable by a scar separating the strip from the nacreous layer of the rest of the shell. This description was applicable to all the mussel samples examined in this study.

Variation in morphotype frequencies between *M*. *edulis* and *M*. *trossulus* within and between contact zones revealed in the study is illustrated in Fig 1, where the estimates of *P(T|edu)* and *P(T|tros)* (i.e. the proportions of T-morphotypes among *M*. *edulis* and *M*. *trossulus*, respectively) in pooled samples from different sets are provided. *P(T|edu)* was 0.53 in the saline Barents Sea (*BH*) and less than 10% in all the other sets. In its turn, *P(T|tros)* was 0.17 in *BALT*, 0.42 in *NORW*, 0.49 in the *GOM* and more than 0.75 in *WSBL* and *SCOT*. *P(T|tros)* estimates in Norway and the Gulf of Maine were much affected by the few outlier samples (see below). If we discard these samples, *P(T|tros)* will make up 0.54 in Norway and 0.71 in the Gulf of Maine.

Fig 1 also shows the morphotype frequencies in putatively pure populations of species sampled at a distance from the contact zones. Within the ancestral range of *M*. *trossulus* in the Pacific, the populations were nearly monomorphic for the T-morphotype. In the Passamaquoddy Bay *P(T|tros)* was 0.81, i.e. close to that in most of *M*. *trossulus* populations in the Gulf of Maine. All reference *M*. *edulis* populations from temperate areas (Long Island Sound and Cape Cod in western Atlantic, Northern and Norwegian Seas in Europe) were nearly monomorphic for the E-morphotype. At the northeast extreme of the species range in eastern Atlantic, in the southwestern Barents Sea, *P(T|edu)* varied considerably between the samples, in particular between the samples from brackish (range 0–3%) and saline (0.35–0.70%) localities (see S2 Table), as it did along the Barents sea coast of the Kola Peninsula. Increased *P(T|edu)* was also recorded in two northernmost samples from western Atlantic, Greenland (0.66) and the Gulf of Saint Lawrence (0.73).

### Genotypic structure of samples

**Fig 2:**
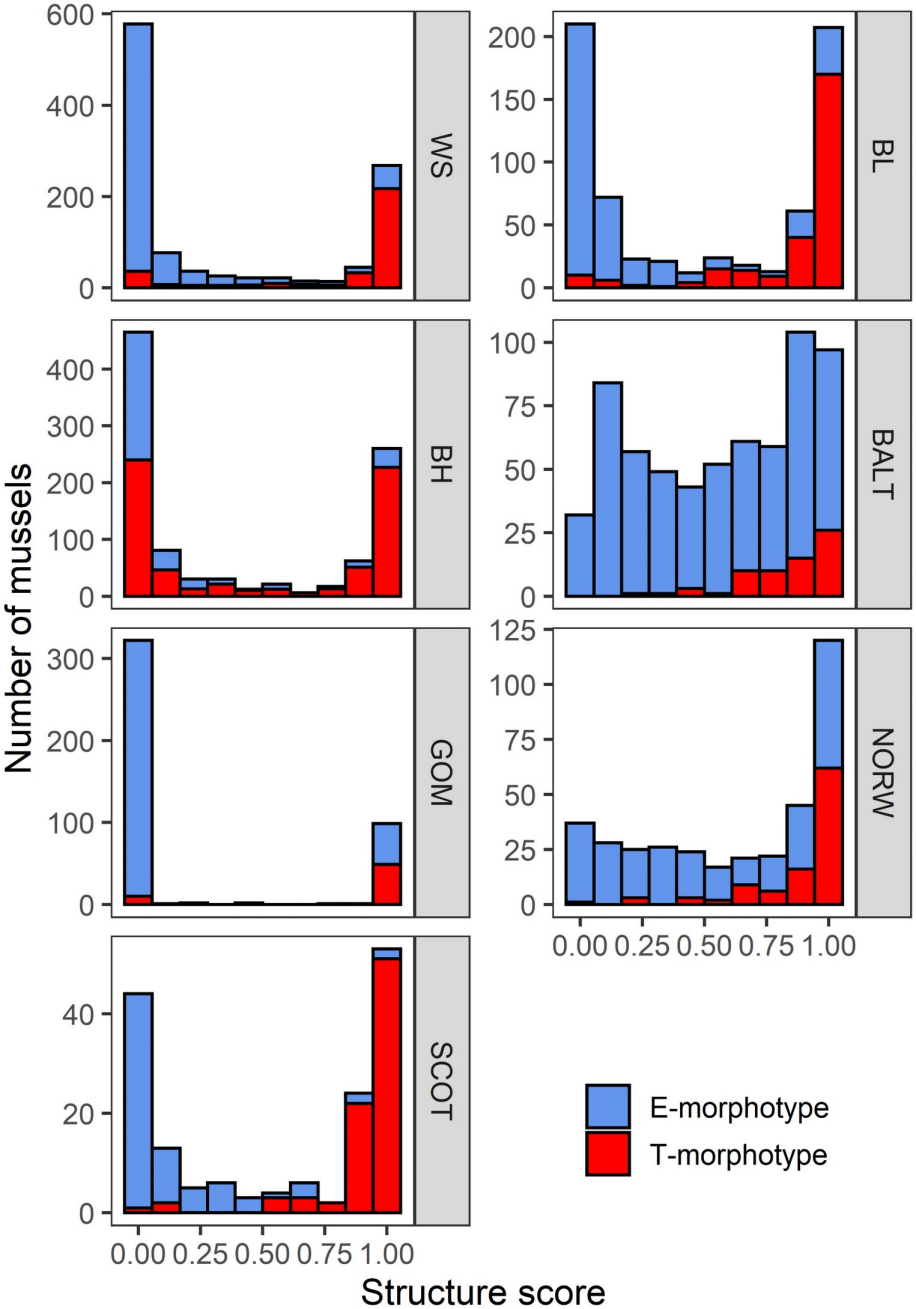
Frequency distributions of individual q-values in pooled samples from contact zones between *M*. *edulis* and *M*. *trossulus*. Numbers of individuals are plotted on the ordinates, with q-values at 10% intervals as abscises. Red and blue bars indicate T- and E-morphotypes, correspondingly.

Distributions of individual q-values in the samples ordinated by the proportion of *M*. *trossulus* (*Ptros*) and the frequency distributions of q-values at 10% intervals (E- and T-morphotypes are indicated) in different contact zones are provided in the S2 Fig and Fig 2, respectively. Distributions were pronouncedly bimodal in *GOM*, *SCOT*, *WS*, *BL* and *BH*, with most of the mussels having low (q<0.2) or high (q>0.8) q-values (putative purebreds of *M*. *edulis* and *M*. *trossulus*, respectively) and less than 15% having intermediate values (putative hybrids). However, the distributions were more flattened in *NORW* and *BALT* (about 30% and 40% of intermediates, respectively). *PT* positively correlated with q-values in each set (Fig 2, S2A Fig). Putative hybrids of T-morphotypes tended to be more common in *M*. *trossulus*-dominated populations (high *Ptros*) than in *M*. *edulis*-dominated ones (S2B Fig).

Relationships between *PT* estimates in the subsamples of mussels with q<0.2 (putative purebreds of *M*. *edulis*) and q<0.5 (genotypes dominated by *M*. *edulis* genes), as well as between subsamples with q>0.8 and q>0.5 (putative purebreds of *M*. *trossulus* and genotypes dominated by its genes) in the samples were close to 1:1 (S3 Fig).

Based on these results, two caveats should be made before embarking upon the analyses based on comparison of “*M*. *trossulus*” (q>0.5) and “*M*. *edulis*” (q<0.5). (1) These categories should be interpreted as admixed genotypes dominated by genes of one of the species in *NORW* and *BALT* and as purebreds in the other contact zones. (2) Morphotype frequencies in hybrids are intermediate between those in parental species, yet in not considering hybrids as a separate class we only slightly bias interspecific differences.

### Associations between morphotypes and genotypes around the Kola Peninsula

**Fig 3:**
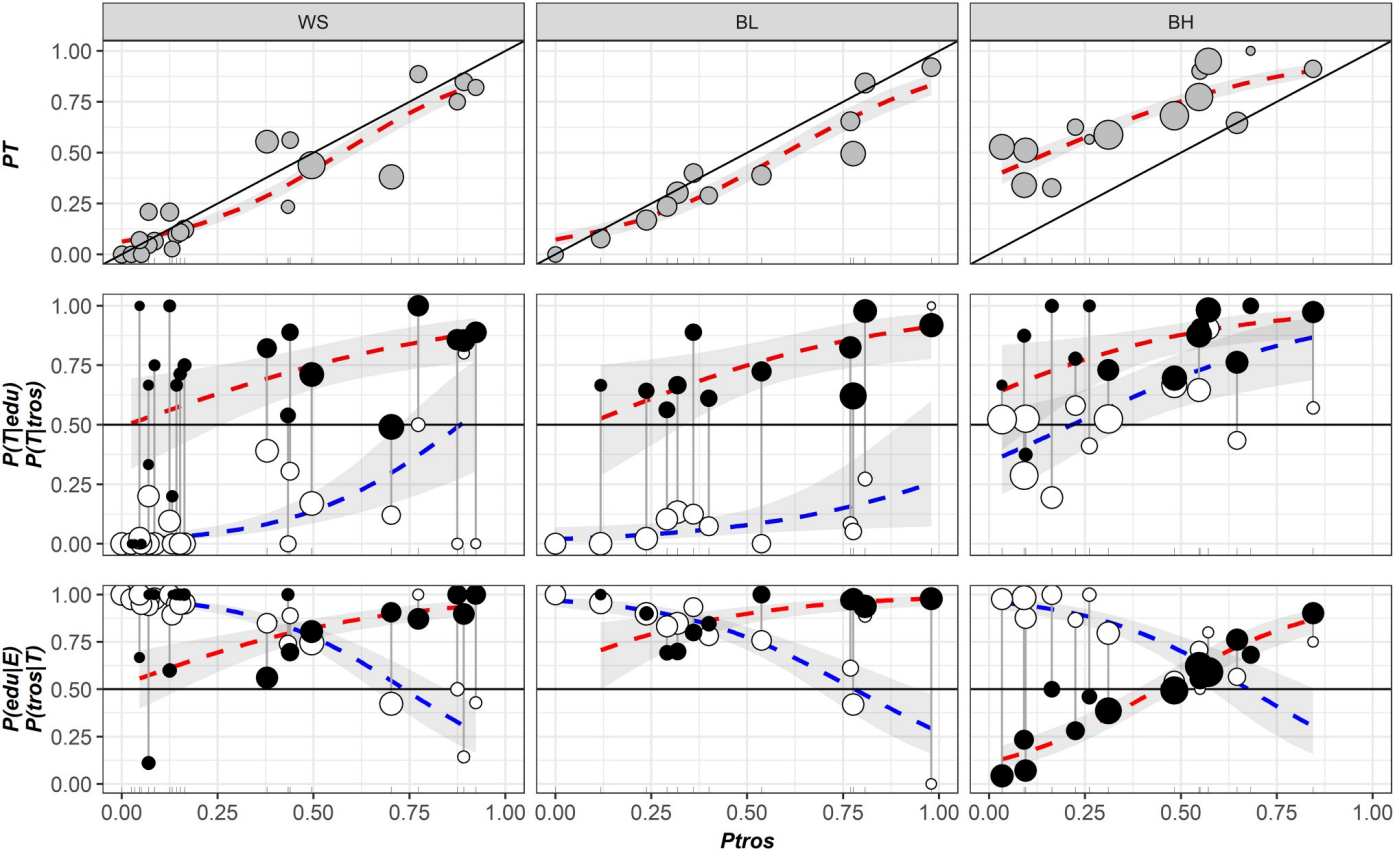
Variation of PT, *P(T|tros)*, *P(T|edu)*, *P(tros|T)*, *P(edu|E)* as functions of Ptros in the White Sea (*WS*), brackish Barents Sea (*BL*) and saline Barents Sea (*BH*). Points–empirical estimates, their size is proportional to sample size (see S1 Table). Lines–regression model predictions, grey filling– 95% confidence intervals of regressions. (A) Proportions of T-morphotypes (*PT*) (Model 1). (B). Proportions of T-morphotypes among *M*. *trossulus* (*P(T|tros)*, filled points) and *M*. *edulis* (*P(T|edu)*, empty points) (Model 2). (C) Frequencies of *M*. *trossulus* among T-morphotypes (*P(tros|T)*, filled points) and of *M*. *edulis* among E-morphotypes (*P(edu|E)*, empty points) (Model 4). Vertical lines on B and C connect subsamples of *M*. *trossulus* and *M*. *edulis* from the same samples.

Variation patterns of *PT*, *P(T|tros)*, *P(T|edu)*, *P(tros|T)*, *P(edu|E)* as functions of *Ptros* in samples from the White Sea (*WS*), the brackish Barents Sea (*BL*) and the saline Barents Sea (*BH*) are visualized in Fig 3. The results of the regression analysis are summarized in S3 Table.

A significant positive association between the proportions of *M*. *trossulus* (*Ptros*) and the proportions of T-morphotypes (*PT*) in samples was revealed for all the three sample sets (Model 1, S3 Table, Fig 3). For *WS* and *BL*, the data points were generally scattered around the Y = X line, while the regression lines approached it closely, indicating a high proportionality between *Ptros* and *PT* and an almost straightforward relationship between these values. For *BH*, the data points were scattered above the Y = X line and the regression line lay higher than the regression lines constructed for *WS* and *BL*. This means that in samples with a similar taxonomic structure, the frequencies of T-morphotypes were always higher in the saline localities in the Barents Sea than in the White Sea and the brackish localities in the Barents Sea.

The analysis of the frequencies of T-morphotypes in subsamples of *M*. *edulis* (*P(T|edu)*) and *M*. *trossulus* (*P(T|tros)*) against proportions of *M*. *trossulus* in samples (*Ptros*) revealed the following patterns (Model 2, S3 Table, Fig 3). There was a universal tendency towards a higher frequency of T-morphotypes among *M*. *trossulus* than among *M*. *edulis*. This tendency was quite strong in *WS* and *BL* (expected differences in morphotype frequencies between species about 0.65 for *Ptros* = 0.5). In *BH* it was rather weak (expected differences 0.18 for *Ptros* = 0.5) due to an increased *P(T|edu)* but significant (confidential intervals for *Ptros* = 0.5 did not overlap, Fig 2). A positive correlation of *P(T|tros)* and *P(T|edu)* with *Ptros* was found in all the three subsets. This means that with the increasing contribution of *M*. *trossulus* to the samples the frequencies of T-morphotypes increased both among *M*. *edulis* and among *M*. *trossulus*.

The probability of correct identification of *M*. *trossulus* by the T-morphotype (the frequency of *M*. *trossulus* among T-morphotypes (*P(tros|T)*) expectedly increased with the increasing *Ptros*, while the probability of correct identification of *M*. *edulis* by the E-morphotype (*P(edu|E)*) demonstrated an opposite pattern (Model 3, S3 Table, Fig 3). In the *M*. *trossulus*-dominated populations, *P(tros|T)* tended to one (any mussel with a T-morphotype is 100% *M*. *trossulus*), while *P(edu|E)* tended to zero (any mussel with an E-morphotype is 100% *M*. *trossulus*), and vice versa. In the well-mixed samples (*Ptros* = 0.5) the predictive values for both species were about 0.75–0.85 in *WS* and *BL* but only 0.60–0.70 in *BH* (Fig 3). It means that the morphotype test has a much lower predictive value in the saline Barents Sea than in the brackish Barents Sea and in the White Sea (the predictive value of 0.5 means a random association between the genotype and the morphotype). It is evident from Fig 3 that a low predictive value of the test in *BH* is mainly due to a generally high frequencies of T-morphotypes in *M*. *edulis P(T|edu)*. The statistical analysis indicates that both *P(tros|T)* and *P(edu|E)* predicted by the model were smaller in *BH* than in *WS* and *BL*.

For each of the GLMM models considered (Model 2 and 3), marginal and conditional pseudoR^2^ were close to each other (S3 Table). This indicates that the role of the random factor (*Sample*) as regulator of models was weak, i.e. the reproducibility of the results in different populations was satisfactory.

In comparisons between sets, the regression coefficients did not differ statistically for *WS* and *BL*, while *BH* was always different from *WS* (S3 Table). To assess the possibility of pooling the data sets, we compared the AIC of Model 3 (AIC = 2175.1) with AICs of three other models based on differently pooled *WS*, *BL* and *BH* sets. The model based on pooled *WS* and *BL* (*WSBL*) and *BH* showed the lowest AIC (AIC = 2172.7). Therefore, in the following analyses we will consider two sets, *WSBL* and *BH*.

### Associations between morphotypes and genotypes around the Atlantic

**Fig 4:**
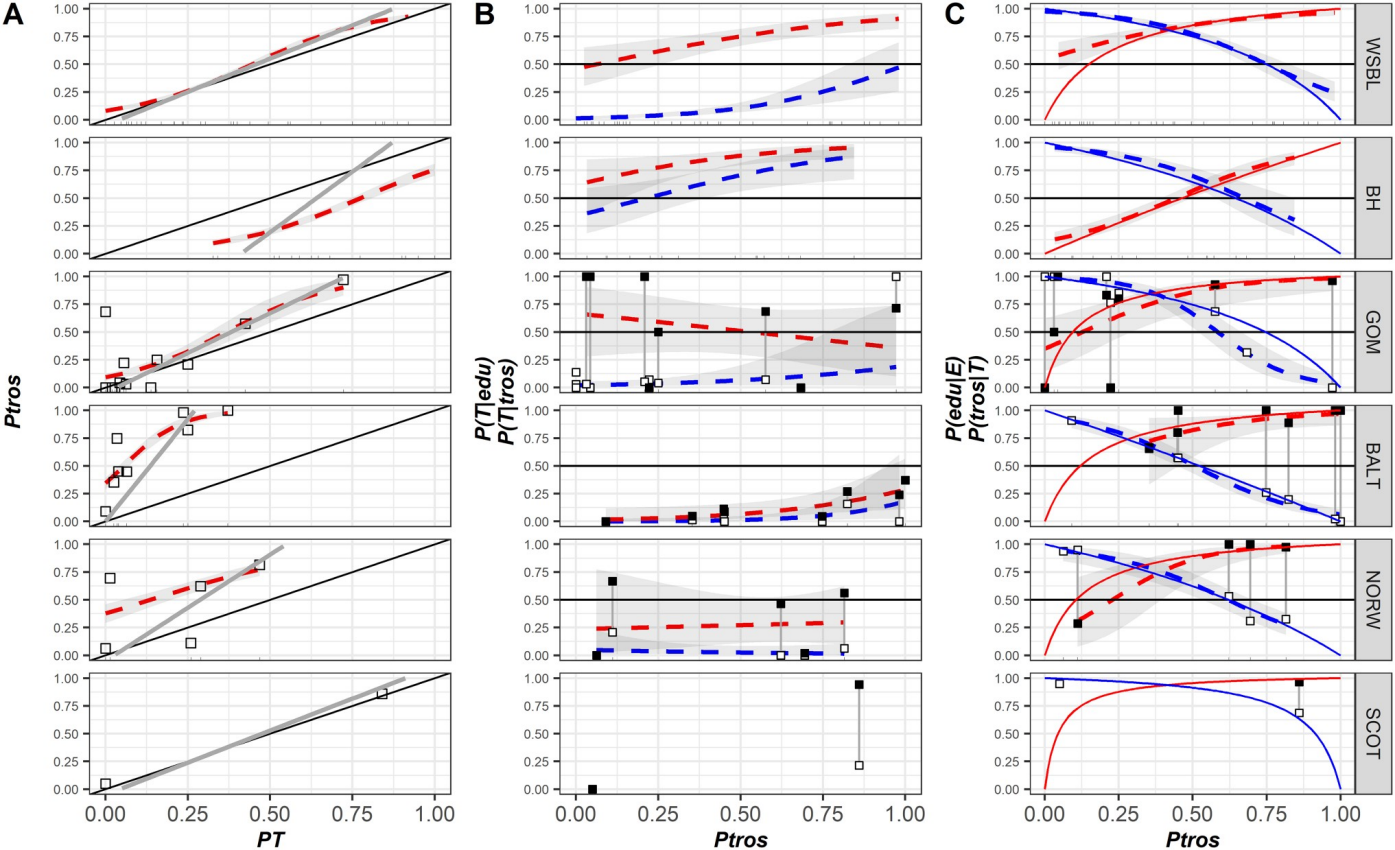
Predictive power of the morphotype test in different contact zones. (A) Dependence of proportion of *M*. *trossulus* (*Ptros*) on proportion of T-morphotypes (*PT*). Dotted lines are empirical regressions (Model 4). Solid gray lines–predictions of “*Ptros* by *PT* calculator” (Eq 3). Solid black lines represent Y = X dependence. (B) Probability to find a mussel with a T-morphotype among *M*. *edulis* (*P(T|edu)*) (empty points), and *M*. *trossulus* (*P(T|tros)*) (filled points) as a function of *Ptros*. Lines are empirical regressions (Model 5). (C) Probability of correct species identification by the morphotype test: *M*. *trossulus* by T-morphotype, *P(tros|T)* (filled points) and *M*. *edulis* by E-morphotype, *P(edu|E)* (empty points) as a function of *Ptros*. Dotted lines are empirical regressions (Model 6). Sold lines–predictions of “genotype by morphotype calculator” for *M*. *trossulus* (Eq 1, red line) and *M*. *edulis* (Eq 2, blue line). On each graph, dots represent the observed proportions in samples, and shaded areas around regression lines– 95% CI of regressions.

The patterns of *Ptros* variation against *PT* and the patterns of *P(T|tros)*, *P(T|edu)*, *P(tros|T)* and *P(edu|E)* variation against *Ptros* in samples from different geographical zones are visualized in Fig 4. The results of the regression analysis are summarized in S3 Table. The Scottish material was not included in the regression analyses. Re-analyses of the data from the White and the Barents Sea (*WSBL* and *BH* sets) together with the data from other regions revealed the same patterns as those described above. Again, in all the cases when mixed models were used (Model 5, Model 6, S3 Table) the marginal and conditional pseudoR2 were close to each other (S3 Table) indicating a weak role of the random factor (*Set*) as regulator of models, i.e. a satisfactory reproducibility of the results from population to population in all the regions.

The proportion of *M*. *trossulus* in samples (*Ptros*) was positively correlated with the proportion of T-morphotypes (*PT*) in the other sets, as it did in the samples from the White and the Barents Sea. This tendency was significant for all the sets (Fig 4; Model 4, S3 Table). Otherwise, the patterns of variation were different for different sets. For *GOM*, the regression line stretched above the Y = X line but close to it, indicating the proportionality between *PT* and *Ptros*. For *BALT*, the regression slope was very steep, and the regression line rapidly diverged from the Y = X line. This was due to the fact that the *PT* range in *BALT* was, unlike the situation in the other sets, very narrow (0–0.4) as compared with the *Ptros* range (~0–1), and the small surplus of T-morphotypes in the samples was accompanied by a strong increase in the *M*. *trossulus* prevalence. A similar tendency was observed in the scanty material from *NORW*. Both *SCOT* samples fell on the Y = X line. Noteworthy are a few “outlier” samples from *GOM* and *NORW*, in which *PT* was close to zero but *Ptros* was high.

While frequencies of T-morphotypes in *M*. *edulis* (*P(T|edu)*) were low everywhere but in *BH*, frequencies of T-morphotypes in *M*. *trossulus* (*P(T|tros)*) demonstrated a strong variation among sets and a noticeable variation within some sets (Fig 4; Model 5; S3 Table). Similarly to *WSBL*, most *M*. *trossulus* had T-morphotypes in *GOM* and *SCOT* but not in *BALT* and *NORW*. For *Ptros* = 0.5, expected differences in the morphotype frequencies between the species were about 0.44 for *GOM*, 0.06 for *BALT* and 0.24 for *NORW*. A significant positive dependence of the frequencies of T-morphotype on *Ptros* among conspecific genotypes, which was so prominent in the White and the Barents Sea, was recorded elsewhere only in *BALT* for *P(T|tros)* (S3 Table).

The pattern of dependence of *P(tros|T)* and *P(edu|E)* on *Ptros* in *GOM*, *BALT* and *NORW* (Model 6. Fig 4, S3 Table) was the same as in the samples from the Kola Peninsula (Model 3. Fig 3, S3 Table): *P(tros|T)* increased with the increasing *Ptros*, while *P(edu|E)* showed an opposite tendency. To simplify and formalize the comparison, we provide the predictions of Model 6 for equally mixed populations (*Ptros* = 0.5) together with their 95% confidence intervals in Table 1, where actual proportions of *M*. *trossulus* among T-morphotypes (*P(T|tros)*) and *M*. *edulis* among E-morphotypes (*P(T|edu)*) in pooled samples from the respected sets are also provided.

**Table 1:** Proportions of *M*. *trossulus* among T-morphotypes (*P(tros|T)*) and proportions of *M*. *edulis* among E-morphotypes (*P(edu|E)*) in pooled samples (direct count) and in equally mixed samples (predictions by the regression Model 6) in different sample sets.

	*P(edu|E)*		*P(tros|T)*	
Set	Ptros = 0.5	In the data	Ptros = 0.5	In the data
WSBL	0.77 (0.73–0.81)	0.86	0.85 (0.82–0.89)	0.86
BH	0.70 (0.61–0.78)	0.84	0.57 (0.51–0.63)	0.48
GOM	0.66 (0.54–0.77)	0.86	0.86 (0.68–0.95)	0.80
BALT	0.51 (0.44–0.58)	0.46	0.82 (0.58–0.94)	0.93
NORW	0.64 (0.53–0.74)	0.51	0.86 (0.68–0.95)	0.93
SCOT	-	0.90	-	0.96

Low and upper boundaries of 95% confidence intervals are provided for predicted values (in brackets).

For equally mixed populations the predictive values of *P(edu|E)* in *BALT* did not differ significantly from 0.5, which corresponds to an equal probability of correct and incorrect identification. At the same time, the probabilities of correct identification of *M*. *trossulus* by the T-morphotype in *GOM*, *BALT* and *NORW* were quite high (for the range of *Ptros*≥0.5). In general, the highest predictive values for both species were revealed in *WSBL*.

Using the coefficients of the regression models Model 4 and Model 6 (S3 Table), we constructed a set of formulas predicting the taxonomic structure (*Ptros*) and the probability of correct species identification (*P(tros|T)*, *P(edu|E)*) using the morphotype test (Table 2). These formulas were further used for the comparison of predictions made with these regression models and the predictions proposed by Eqs 1, 2 and 3.

**Table 2:** Formulas used for taxonomic and individual assignment using morphotype tests in different sample sets accordingly to the regression model coefficients represented in S3 Table.

Region	Model 4	Model 6 E-morphotype	Model 6 T-morphotype
WSBL	$Ptros=\frac{e^{-2.4+5.4PT}}{1+e^{-2.4+5.4PT}}$	$P(edu|E)=\frac{e^{3.7-4.9Ptros}}{1+e^{3.7-4.9Ptros}}$	$P(tros|T)=\frac{e^{0.2+3.2Ptros}}{1+e^{0.2+3.2Ptros}}$
BH	$Ptros=\frac{e^{-3.9+5.0PT}}{1+e^{-3.9+5.0PT}}$	$P(edu|E)=\frac{e^{3.3-4.8Ptros}}{1+e^{3.3-4.8Ptros}}$	$P(tros|T)=\frac{e^{-2.1+4.7Ptros}}{1+e^{-2.1+4.7Ptros}}$
GOM	$Ptros=\frac{e^{-2.3+6.2PT}}{1+e^{-2.3+6.2PT}}$	$P(edu|E)=\frac{e^{4.7-8.1Ptros}}{1+e^{4.7-8.1Ptros}}$	$P(tros|T)=\frac{e^{-0.6+4.9Ptros}}{1+e^{-0.6+4.9Ptros}}$
BALT	$Ptros=\frac{e^{-0.6+11.6PT}}{1+e^{-0.6+11.6PT}}$	$P(edu|E)=\frac{e^{2.7-5.4Ptros}}{1+e^{2.7-5.4Ptros}}$	$P(tros|T)=\frac{e^{-0.4+3.9Ptros}}{1+e^{-0.4+3.9Ptros}}$
NORW	$Ptros=\frac{e^{-0.5+3.7PT}}{1+e^{-0.5+3.7PT}}$	$P(edu|E)=\frac{e^{3.1-5.0Ptros}}{1+e^{3.1-5.0Ptros}}$	$P(tros|T)=\frac{e^{-1.5+6.3Ptros}}{1+e^{-1.5+6.3Ptros}}$

### Associations between morphotypes and shell size

There was no clear statistical relationship between the size and the morphotype of conspecific mussels. At the level of individual samples, the probability of finding a T-morphotype increased with the mussel size (a positive slope-term of the regression) in 16 out of 34 informative comparisons (when species-specific genotypes were both present and polymorphic for morphotypes) for *M*. *edulis* and in 17 out of 43 comparisons for *M*. *trossulus*. The slope-terms of the regression models were individually significant (p<0.05) in four cases for *M*. *edulis* and in four cases for *M*. *trossulus*, but only in one case when the correction for multiple testing was applied (sample Berg05, see S4 Table). We also checked for the presence of any patterns in residuals from Model 6 as a function of mussel size but none was found.

#### Prediction of taxonomic structure of populations and predictive values of the morphotype test based on probability calculators

We applied Eqs 1 and 2 (“genotype by morphotype calculator”) and Eq 3 (“*Ptros* by *PT* calculator”) using as an input for assessment of equations parameters (*P(T|tros)*, *P(T|edu)*) the data on all possible pairs of samples from *WSBL* and compared the values predicted by these equations with those predicted by regression Models 6 and 4, respectively (Table 2).

**Fig 5:**
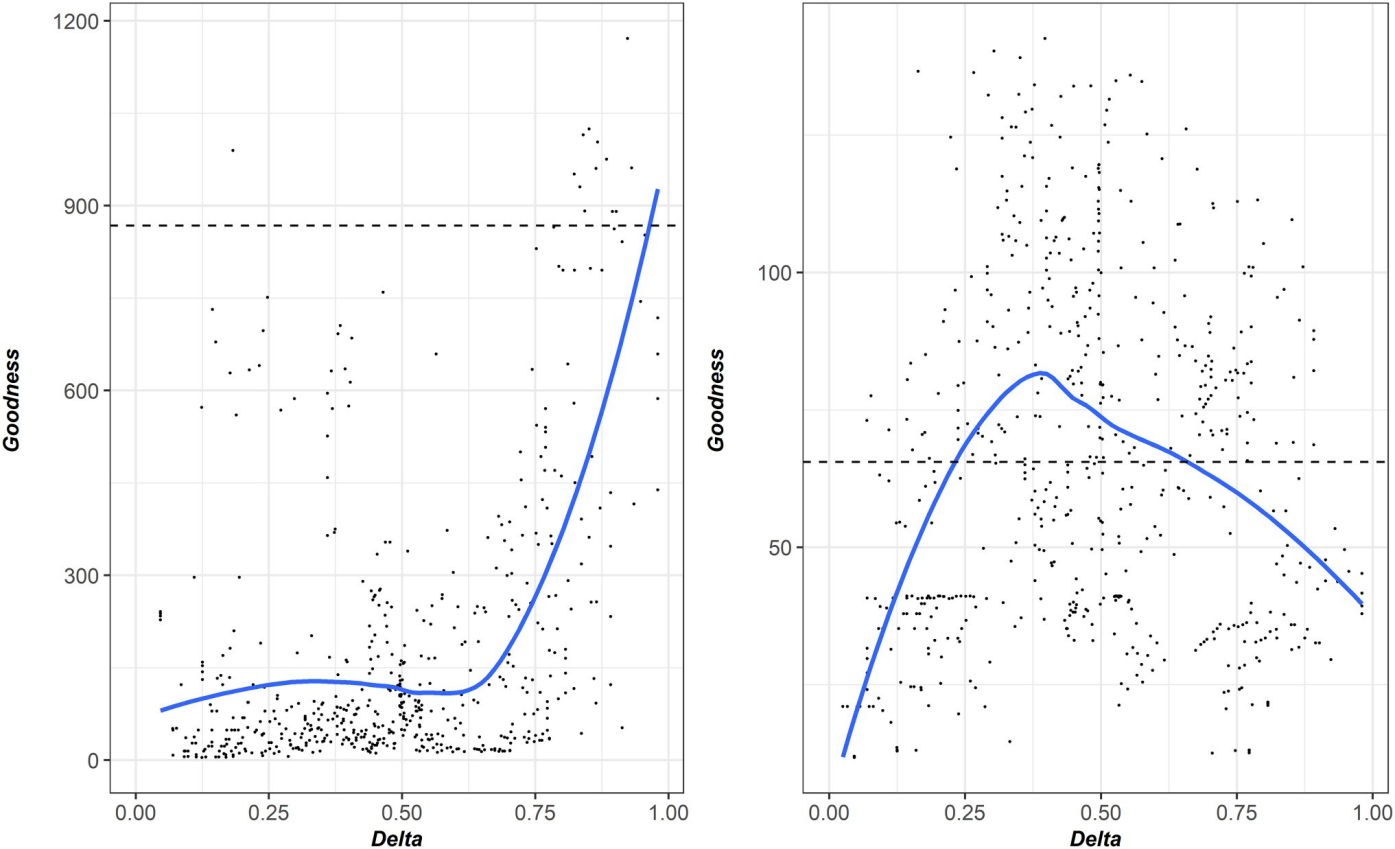
Correspondence between “*Ptros* by *PT* calculator” (Eq 3, left graph) and “genotype by morphotype calculator” predictions (Eqs 1 and 2, right graph) and regression Model 6 and Model 4, respectively. Each point corresponds to a unique pair combination of samples from *WSBL* set. OX axis reflects dissimilarity of genetic structure in each pair (Delta) (for pure conspecific samples Delta takes a value of zero, for equally mixed samples– 0.5, for two pure heterospecific samples– 1). OY: goodness of correspondence between assessment of predictive values by equations and regression models.

Fig 5 illustrates the goodness of correspondence of the two predictions depending on the genetic constitution of the paired samples as expressed by the Delta index. The best predictions of *Ptros* were obtained when the most dissimilar samples consisting of nearly pure *M*. *edulis* and *M*. *trossulus* (Delta >0.75) were taken for Eq 3 calibration. The best predictions of *P(edu|E)* and *P(tros|T)* values were obtained when the most mixed samples (*Ptros* of both samples close to 0.5; range of Delta 0.25–0.5) were taken for Eqs 1 and 2 calibration.

Therefore, in order to predict *Ptros* using “*Ptros* by *PT* calculator” one should use the most dissimilar samples to assess *P(T|edu)* and *P(T|tros)* as calculator parameters. In order to predict *P(edu|E)* and *P(tros|T)* using “genotype by morphotype calculator” one should assess the parameters using the most mixed samples. However, the *Ptros* as input in Eqs 1 and 2 should be calculated as in the previous case i.e. using the most dissimilar samples and Eq 3. To illustrate the approach (see Fig 4) for *WSBL*, *BH*, *GOM* and *BALT* we used pooled sets of samples with *Ptros* <0.1 and >0.8 to calibrate “*Ptros* by *PT* calculator” and 0.45 < *Ptros* < 0.65 to calibrate “genotype by morphotype calculator” as described above (samples included are indicated in S1 Table). We used pooled but not individual samples to avoid basements due to small sample size. The given ranges of *Ptros* were used because of the lack of *M*. *trossulus*-dominated samples in most sets. For *NORW* and *SCOT* we pooled all the samples because of the lack of data.

Visual inspection of Fig 4A revealed a nearly ideal correspondence between regression lines and predictions of “*Ptros* by *PT* calculator” in the case of *WS*BS and *GOM*. In the case of *SCOT*, where only two samples were available, the line derived from Eq 3 approached the Y = X line. A rather close though not ideal correspondence was observed in the case of *BALT*, deviation being due to a very high slope term of the regression. *Ptros* was slightly underestimated by the calculator in this case. The worst correspondence between Eq 3 and Model 4 was observed in the case of *NORW* and *BH*. In *BH Ptros* was severely overestimated by the calculator, which was opposite to the situation in *BALT*. In *NORW* both regression and predictions of calculator were severely affected by the outlier sample.

As for the “genotype by morphotype calculator”, the predictions generally were in good correspondence with the regression lines (calculator’s lines were within 95% CI of regressions). Deviations were observed for *P(tros|T)* predictions in *WSBL* for *Ptros*<0.25 and *P(edu|E)* in *GOM* for *Ptros*>0.6 i.e. in the *Ptros* ranges corresponding to a small prevalence of the species.

An exercise with the “lazy *Ptros* by *PT* calculator”, in which the highest and the lowest *PT* in samples from regional sets are used as *P(T|tros)* and *P(T|edu)* parameters of Eq 3, had the following results (S4 Fig). In *WS*, *BL* and *GOM* correspondence between the observed and the predicted *Ptros* values in samples was generally good. In *BH*, *Ptros* was slightly overestimated by the calculator due to the absence of pure *M*. *trossulus* samples in the data and the formal choice of a numerically small (N = 22; see S1 Table) sample with the highest *PT* but not the highest *Ptros* as the “calibrating” one. For *BALT* and *NORW* discrepancies were much stronger, the reasons being the same as in case of “*Ptros* by *PT* calculator” (see above).

## Discussion

In some study areas, such as community ecology, biomonitoring and aquaculture, the knowledge about the taxonomic structure of blue mussel populations and a rough classification of individuals into “species” may be more valuable than the precise information about the genetic affinity (e.g. Structure q-value) of any given mussel. In the light of this, our finding that *M*. *edulis* and *M*. *trossulus* genotypes in the White Sea differed by the frequencies of the shell morphotypes [16] seemed very promising. It gave hope that this knowledge could be obtained for these species by a quick examination of the inner side of the shells, without genotyping, which is expensive, time-consuming and requires soft tissues (genotyping of shell material is possible [43, 44] but is not yet routine practice). In this study we reanalyzed abundant data from [16] and derived robust relationships between the proportions of the morphotypes in the populations and their taxonomic structure as well as between the proportions of the morphotypes in populations and the probabilities of mussels of different morphotypes being *M*. *trossulus* and *M*. *edulis*. These relationships could be used for a reliable prediction of the taxonomic structure of any population in the White Sea. Moreover, any mussel in an equally mixed population could be identified as *M*. *trossulus* or *M*. *edulis* with the accuracy of about 80% (a bit less than it was predicted basing on frequencies of the morphotypes in pooled data on the White Sea *M*. *edulis* and *M*. *trossulus*, see Introduction). With the increasing imbalance between the species (and hence the morphotypes) in a population, the identification of the dominant species became more reliable though the identification of the minor species became less so.

The ultimate goal of our study was to find out whether the possibility of identifying *M*. *edulis* and *M*. *trossulus* by the morphotype was a “privilege” of the researchers working at the White Sea or whether this approach could be used for identification of these two species worldwide. Though our data on the contact zones between the species outside northern Russia were limited, our results indicate that this approach may be useful everywhere since interspecific differences in the morphotype frequencies were ubiquitous and unidirectional. However, its utility is evidently different for different contact zones.

An evident limitation of the morphotype test in the contact zones with extensive hybridization such as the Baltic Sea and Western Norway is that hybrids, which are quite numerous and ecologically important, are considered together with the closest purebreds. At the same time, since hybrids are intermediate in morphotype frequencies between the species [[16]; this study], their numbers cannot not affect the test’s predictions of the taxonomic structure of populations in any contact zone.

Though the hypotheses that different mussel species may differ by the extent of the nacreous layer development under the ligament nympha was already suggested by Zolotarev, Shurova [45] and Vervoenen et al. [46], the morphotypes were actually applied to species identification by Khaitov et al. [22]. Here we show that the morphotype test is a promising tool. Once it has been evaluated, i.e. associations between morphotypes and species-specific genotypes have been worked out at the individual and the population level, it will hopefully deserve more attention from the blue mussel researchers.

To note, another method for a fast morphological diagnosis of *M*. *trossulus* and *M*. *edulis* was suggested by Beaumont et al. [19], who showed that commercially damaging “fragile mussels” in Scottish *M*. *edulis* plantations were genetically similar to *M*. *trossulus*. The fragile mussels differed from *M*. *edulis* (and the reference *M*. *galloprovincialis*) by a combination of shell traits including shape, the degree of expression of growth ridges and the color of the inside. The promising identification method was however not developed further. A comparison of the photographs of shells in Beaumont et al. [19] with our Barents Sea samples (S1E Fig) shows that the interspecies differences in the Barents Sea are less striking than in Scotland.

We will start with the discussion of the patterns of variation of the morphotype frequencies revealed in our study. Then we will discuss the applicability of the morphotype test in different contact zones. In the closing section, the limitations of single-marker taxonomic tests for blue mussels and other taxa will be outlined.

### Variation of morphotype frequencies among conspecific populations

Some variation in the morphotype frequencies was observed among putatively pure conspecific populations sampled at a distance from the contact zones. Samples of pure *M*. *edulis* from the temperate seas (i.e. all except those from the eastern Barents Sea and Greenland) were nearly monomorphic for the E-morphotype, while the northern samples were more polymorphic and diverse. In turn, the reference populations of *M*. *trossulus* from the northwestern and northeastern Pacific (Washington) were nearly monomorphic for the T-morphotype. Nevertheless we cannot necessarily exclude the possibility of geographic variation in *M*. *trossulus* in its ancestral Pacific range or confirm that the T-morphotype is the “ancestral” state for this species. Zolotarev [47] identified morphotypes in small samples of genotyped mussels (from [7]) and found elevated frequencies of the E-morphotype in *M*. *trossulus* from Oregon (northeastern Pacific).

In *M*. *trossulus* the morphotype frequencies varied between the contact zones, and elevated frequencies of E-morphotypes were found in Norway and, especially, in the Baltic Sea. The variation within contact zones was mostly due to the few “outlier” samples from the Gulf of Maine and Norway. On the contrary, *M*. *edulis* showed little variation between zones, the T-morphotype being universally rare. In a notable exception the T-morphotype frequency was clearly elevated (up to 40%) in samples from saline localities (> 30 ppt) in Kola Bay and surroundings. Similar salinity-related variation was found in *M*. *edulis* from the more eastern areas of the Barents Sea, at some distance from the contact zone between these species along the Kola Peninsula coast.

Finally, an analysis of the abundant material from the White and the Barents Sea demonstrated how the morphotype frequencies varied with the taxonomic composition of the mixed populations. The frequencies of the T-morphotype increased both among *M*. *edulis* and among *M*. *trossulus* genotypes with the increasing prevalence of *M*. *trossulus* in the samples.

### Unusual features of *M*. *trossulus* from Norway and the Baltic Sea

*M*. *trossulus* from the Baltic Sea and Norway were characterized by extremely high frequencies of the E-morphotype. Two hypotheses, which are not mutually exclusive, can be offered to explain this phenomenon. One hypothesis links the morphotypes to species-specific genes that can introgress between species as a result of extensive hybridization and backcrossing. The Baltic *M*. *trossulus* is stronger introgressed by *M*. *edulis* genes than any other Atlantic population [3, 33]. Due to its mixed genetic nature, the Baltic mussel is sometimes considered as a unique *M*. *edulis* x *M*. *trossulus* hybrid swarm, which is fundamentally different from the “oceanic” *M*. *trossulus* [3]. While the genetic data from Norway are limited, it is evident that the local Norwegian *M*. *trossulus* populations can be strongly introgressed by *M*. *edulis* genes too [48].

In the second hypothesis, the frequency of the T-morphotype in *M*. *trossulus* is reduced under the influence of some environmental factors, both micro- and macro-geographical. We suspect that the nearly zero frequencies of the T-morphotype in the “outlier” samples (one from Norway, almost from the same place as the other Bergen samples, and two from Cobscook Bay in the Gulf of Maine [CBCP, CBSC in S1 Table]) could be explained by the impact of some cryptic local factors, though a more prosaic explanation such as the mislabeling of mussels in the collections cannot be entirely ruled out.

### Salinity-related variation in *M. edulis*

While local factors putatively affecting morphotype frequencies in *M*. *trossulus* remained cryptic, in the Barents Sea we managed to identify one such factor governing morphotype frequencies in *M*. *edulis*: salinity or a factor/factors linked to salinity. The eastern part of the Barents Sea, where this variation was evident, is also the coldest. The border between the more temperate populations of *M*. *edulis* with “normal” (high) frequencies of the E-morphotype and the more Arctic populations with lower frequencies of the E-morphotype in oceanic habitats runs somewhere between North Cape and the Kola Bay (Fig 1). This area has mean annual, summer and winter sea surface temperatures of about 6°C, 9°C and 4°C, respectively (http://esimo.oceanography.ru/).

The functional significance of the morphological character underlying the E- and the T- morphotype (the presence/absence of the nacreous layer under the ligament) is unclear. However, we suspect that the morphotypes might differ in conspecifics by the degree of development of the nacreous layer itself and thus in the thickness and strength of the shell. The nacreous shell layer is mechanically the strongest [49].

Can we expect the shell thickness and structure to differ in mussels from saline (oceanic) and brackish (estuarine) environments in the Arctic? Apart from the low temperatures, the Arctic Sea is characterized by a reduced concentration of calcium carbonates [50] and, seasonally, by low concentrations of planktonic algae, which the mussels feed on [51]. Estuarine habitats are generally characterized by the lowest saturation of carbonates but the highest concentrations of food (seston), which is due to the riverine discharge [52]. This is exemplified by the highest biomasses of Mytilus in estuaries in the Barents Sea [53] and elsewhere [12]. Mussels need both calcium carbonates and energy for shell growth and maintenance. In estuaries, the nacreous layer of the mussel shell is prone to dissolution and corrosion [54] but the mussels can still keep their shells strong if the food is sufficient [52, 54]. If the food is limited, the energy is likely to be allocated to the maintenance of the somatic mass rather than the conservation of the shell ([54] and references therein).

Our hypothesis explaining the assumed differences in the degree of the nacreous layer development between *M*. *edulis* from the brackish and the saline localities in the Arctic is that in the estuaries the mussels allocate more energy for shell maintenance thus keeping their nacreous layer thick while in less corrosive but more famished oceanic habitats they allocate more energy for somatic growth keeping their nacreous layer thin. As a result, the majority of *M*. *edulis* from the saline localities has the undeveloped nacreous layer. It is noteworthy that in the areas where *M*. *edulis* demonstrated salinity-related variation, the morphotype frequencies in *M*. *trossulus* varied negligibly. This could be attributed to a generally lower shell plasticity in “oceanic” (non-Baltic) *M*. *trossulus* than in *M*. *edulis* in response to the environmental stressors ([55], see [22] for more discussion).

Noteworthy, reduced frequencies of the E-morphotype were revealed not only in the eastern Barents Sea but also in northernmost populations of *M*. *edulis* from Greenland and the Gulf of Saint Lawrence in western Atlantic (Fig 1). This indicates that this is an Arctic phenomenon. Unfortunately, the salinity in the sampling localities in the latter two areas is unknown.

### Variation with the taxonomic structure

A positive correlation of the T-morphotype frequencies both in *M*. *edulis* and *M*. *trossulus* with the prevalence of *M*. *trossulus* in the representative data sets from the White and the Barents Sea was to be expected, bearing in mind that *M*. *edulis* and *M*. *trossulus* genotypes are defined by the dominance of the conspecific genes in multilocus genotypes. Hence both genotypes included purebreds as well as some hybrids that are intermediate in morphotype frequencies between purebred *M*. *edulis* and *M*. *trossulus* but usually closer to species dominating the population [[16]; this study]. This means that in our analyses “*M*. *edulis* genotypes” from *M*. *trossulus*-dominated populations included mostly hybrids with an increased frequency of the T-morphotype as compared to the “*M*. *edulis* genotypes” in *M*. *edulis*-dominated populations. In turn, “*M*. *trossulus* genotypes” from *M*. *edulis*-dominated populations included mostly hybrids with a decreased frequency of the T-morphotype as compared to such genotypes in *M*. *trossulus*-dominated populations. This is the cause of the observed unidirectional variation in the morphotype frequencies among *M*. *edulis* and *M*. *trossulus* genotypes with the changing taxonomic structure of populations. To note, the variation of sensitivity and specificity of clinical diagnostic tests with the changing disease prevalence is often observed [56]. For instance, a patient population with a higher disease prevalence may include more severely diseased patients, and the test would consequently perform better [56].

### Applications of the mussel morphotype test

The morphotype test can be universally applied as an alternative to genotyping in three fields. Firstly, it can be used for monitoring the species composition of commercial and wild populations, in particular those used in the “mussel watch” contaminant monitoring programs, because deviations of the morphotype frequencies may be a warning sign of the taxonomic change. Secondly, it may prove useful for mapping the species distributions. Detailed mapping is likely to require a great number of samples because the distribution of the species in contact zones is usually highly mosaic (see [16] and references therein). Thirdly, the morphotype test can be used when only dead mussel shells are available, e.g. for interpretations of the taxonomic structure of natural history collections or samples of dead shells left behind by some mussel predators.

#### Identification of taxonomic structure of populations from contact zones

A reliable application of the morphotype test requires good genotyped references. Ideally, empirical relationships should be established between the morphotype frequencies and the taxonomic structure of populations in a given contact zone, as was done in our study (Table 2). Even our regressions require further refinement for all the contact zones except northern Russia, since they are based on a relatively small number of samples. If such an effort is undertaken for Greenland and high latitude American populations, salinity and trophic conditions should be considered as a potential covariates of the morphotype variation.

The relationships between the morphotype frequencies and the taxonomic structure of populations will have to be established *de novo* in understudied or, potentially, new contact zones. Should the genotyping of more than a few samples covering the range of the morphotype frequencies prove impractical, the relationships could be approximated using the data on at least two genotyped samples with the most contrasting structure (ideally, pure *M*. *edulis* and pure *M*. *trossulus*) and the “*Ptros* by *PT* calculator” (Eq 3) (cf. Fig 3). At the very least, the relationships could be weighed roughly without any genotyping, by taking the minimal and the maximal morphotype frequencies in regional populations as hypothetical corresponding frequencies in pure *M*. *edulis* and pure *M*. *trossulus* populations (“the lazy *Ptros* by *PT* calculator”, cf. S4 Fig). Naturally, such predictions should be treated with the greatest caution.

In case of historical or archaeological collections, the only way to translate the proportion of the T-morphotypes in the samples into the taxonomic structure is to resort to the actualistic principle. If the correspondence between the morphotypes and the genotypes was assessed in the area of the sample origin, one can use this information for retrognosis. Unfortunately, the morphotype test is unlikely to be useful for the interpretation of paleontological data since the morphotype frequencies in conspecifics are affected both by geography and by the local oceanographic conditions, which are variable at a large time scale.

#### Individual identification

The possibility to identify individual mussels by the morphotype seems to be the “privilege” of researchers working at the White Sea and brackish environments of the Barents Sea. The morphotype test also seems to be promising for individual assignment in the Gulf of Maine, except in the outlier samples (see above) and, possibly, in Scotland (unfortunately, the Scottish populations were represented in our analysis only by two samples). In the Baltic Sea and Norway the morphotype test worked reliably only for *M*. *trossulus*-like mussels (to remind, due to an unusually extensive hybridization in these zones, “*M*. *edulis*” and “*M*. *trossulus*” genotypes as defined here include many hybrids), while in the saline areas in the Barents Sea it did so only for *M*. *edulis* mussels.

We would like to stress that, if one plans to use the morphotype test for individual assignment, reliable genetic references are indispensable. These could be either empirical relationships between the proportions of the morphotypes in the samples and the probabilities of mussels of different morphotypes being *M*. *trossulus* or *M*. *edulis* or control genotyping of mussels from the populations of interest. Still, it is noteworthy that the accuracy of individual identification of mussels could be approximated basing on the morphotype frequencies in three “calibration” samples (those with the maximum, the minimum and the intermediate proportions of species) and Eqs 1–3 (cf. Fig 4).

#### Pitfalls of the morphotype test

The morphotype test comes with pitfalls. One of the evident risks is an underestimation of *M*. *trossulus* by morphotypes in some populations, such as those in Norway and the Gulf of Maine, which were the sources of the “outlier” samples. Another is the bias generated by a non-random association of morphotypes with size (or age) of conspecific mussels such as was observed in very rare (about 2%) samples. A further risk are uncertainties in the application of the test to populations from intermediate salinities (about 30 ppt) in the Barents Sea.

### Uses and abuses of single marker taxonomic tests

Traditional species identification relies on diagnostic (fixed) morphologic traits of the organism, usually included in the diagnosis. In the terms of the probability theory, it means that the probability of an individual with a species-specific diagnostic marker being a representative of the species in question is equal to one: *P(species|trait)* = 1. However, in practice the probability can be lower for two reasons. First, because of scoring errors related to the researcher’s skills or the defective condition of the specimen. Second, for the ambiguity in the diagnosticity of a trait. It is generally impossible to determine whether diagnostic characters are indeed fixed if the sample size is finite [57]. Hence, in practice, for diagnostic markers *P(species|trait)* ≤ 1.

Some taxa, however, lack diagnostic characters and have to be identified on the basis of semi-diagnostic ones. This is the case with the blue mussels [7]. In case of semi-diagnostic traits, the researchers do not identify the species of a given individual but assess the probability of its assignment to one or another species. For these traits, P(species|trait) < 1. Similarly, dealing with population assessment we assess the probabilities of finding the representatives of one or another species in a sample but not the true proportion. The most critical point is that P(species|trait) is not constant but varies, yet in predictable manner, with the prevalence of a species in a range [0;1].

A correct application of tests based on semi-diagnostic markers, such as clinical diagnostic tests, ultimately requires a “reference standard” used for verification of the index test results [24]. In our case study of the blue mussels, we used as references the groups of multilocus genotypes (from 4 to 171 645 loci depending on the geographical sample set) defined by the dominance of alleles characteristic of one or the other species. These groups did not represent true species. They included hybrids, some of which (e.g. first- and second generation hybrids) were assigned into groups randomly. To note, multilocus genotyping is seldom employed for identification of cryptic mussel species. Most studies rely on singular or few “standard” diagnostic PCR-based markers, usually nuclear Me15/16 and ITS and mitochondrial COI or 16S markers [11]. Offering the morphotype test as a rough but cost-efficient alternative to genotyping, we have to assess its reliability as compared to single- and few locus tests. It has been long known that the efficiency of “diagnostic” markers for discrimination between *M*. *edulis* and *M*. *trossulus* is different in contact zones in western Atlantic (i.e. the Gulf of Maine) and the Baltic Sea [1]. In the Northwest Atlantic the species are nearly fixed for alternative alleles at Me15/16, ITS and mitochondrial markers, while in the Baltic Sea intraspecific differences at these loci are 20%, 32% and 0%, respectively, due to a introgression of *M*. *edulis* genes into the local *M*. *trossulus* genome [1]. For comparison, the differences in morphotype frequencies between species in the Gulf of Maine and the Baltic Sea are 44% and 15%. As far as we know, the efficiency of taxonomic tests based on singular or a few “standard” loci has not been carefully evaluated for other *M*. *edulis*–*M*. *trossulus* contact zones, though some attempts have been made (see [3, 58]). The recent population genomic studies of hybridizing *Mytilus* species indicate that these species can altogether lack fixed genetic differences due to ubiquitous introgression and that loci can introgress in unpredictable manner in different contact zones [33, 59]. On these grounds, the conventional approach to mussel species identification based on singular molecular markers has been criticized [59]. We do not claim that the morphotype test would fare better than most single-locus taxonomic tests in any contact zone between *M*. *edulis* and *M*. *trossulus*. At the same time, we would like to point out that the performance of these genetic tests has not been evaluated in most contact zones, while that of the morphotype test has been.

A situation when one has to rely on a singular “informal” semi-diagnostic character to distinguish morphologically such old evolutionary lineages as *M*. *edulis* and *M*. *trossulus* is certainly uncommon in taxonomy. At the same time, it is not unique. There are other taxa, which lack fixed diagnostic morphological characters and are identified by semi-diagnostic traits, individual or complex such as like the coordinates of multifactorial analysis. These taxa include subspecies defined according to the 75% rule [60], cryptic species with statistical differentiation [61] and hybridizing species that secondarily lost fixed differences due to introgressive hybridization [62]. Therefore, we hope that our experience of dealing with a non-fixed taxonomic character would be interesting not only to our colleagues working with blue mussels but also to the researchers who study sympatric taxa with vague morphologies and semi-isolated gene pools.

## Supporting information

S1 Fig Variation in the manifestation of mussel morphotypes. A-D. Stereoscopic micrographs of the ligament area of mussel valves. Note that scale bars differ between A-C and D. Strip of the prismatic layer under the ligament nympha is indicated by arrows. A, B. E-morphotypes: the space under the ligament nympha is totally (A) or partially (B) covered by the nacre. C, D. T-morphotypes: a strip of uncovered prismatic layer under the ligament nympha is dark and wide (C; typical of most examined populations) or pale and narrow, recognizable by a scar separating it from the nacreous layer (D; typical of the Gulf of Maine populations). E. External and internal features of the shell valves of *M*. *trossulus* (right) and *M*. *edulis* (left) genotypes from the Kola Bay (from sample Sev.17 in S1 Table). In most cases T-morphotypes (marked by *) and E-morphotypes could be distinguished by an unaided eye.
(TIFF)

S2 Fig Genotypic structure of mussel samples from contact zones between *M*. *edulis* and *M*. *trossulus*. A. Frequency distributions of individual q-values in pooled samples. Red and blue bars indicate T- and E-morphotypes, correspondingly. B. Distributions of individual q-values in samples ordinated by *Ptros* (proportion of *M*. *trossulus*). Red and blue dots indicate T- and E-morphotypes, correspondingly. To avoid overplotting, the horizontal position of all points (individual mussels) was jittered by adding a small random value. For visual purposes, chart areas with maximal dots density are contoured and the probability of T-morphotype presence is shown by the color gradient. The contour lines represent the kernel density estimations (Venables, Ripley 2002) with density2d() function in “ggplot2” package. The probability was assessed using the binomial general additive model, GAM (Zuur, 2012) with the binary outcome (T vs E morphotype) as dependent variable and “Structure q-score”, “*Ptros*” and “*Set*” as independent predictors. *SCOT* and *NORW* were not included in GAM due to poore cover of *Ptros* axis. References for S2 Fig: Venables, W. N. and Ripley, B. D. (2002) Modern Applied Statistics with S. Fourth edition. Springer. Zuur, A.F. (2012) A Beginner’s Guide to Generalized Additive Models with R. Highland Statistics Ltd, Newburgh.
(TIF)

S3 Fig Frequencies of T-morphotypes among mussel genotypes dominated by genes of *M*. *edulis* (q<0.5, *P(T|edu)*) and *M*. *trossulus* (q>0.5, *P(T|tros)*) and putative purebreds of this species (q<0.2 and q>0.2, respectively) in individual samples from contact zones. A. *M*. *edulis*. B. *M*. *trossulus*. Samples from different zones are shown in different colors.
(TIF)

S4 Fig Correspondence between empirical estimates of *Ptros* in samples and predictions of the “lazy *Ptros* by *PT* calculator” (the highest and the lowest *PT* in samples from regional sets are used as *P(T|tros)* and *P(T|edu)* parameters of Eq 3). Dots–estimates, solid line–linear regression, dashed line–Y = X line.
(TIF)

S1 Table Information about samples from contact zones between *M*. *edulis* and *M*. *trossulus*. (XLSX)

S2 Table Information about samples from putatively pure populations of *M*. *edulis* and *M*. *trossulus* out of the contact zones. (XLSX)

S3 Table Parameters of the fitted regression models. (PDF)

S4 Table The probability of the presence of the T-morphotype as a function of mussel size in subsamples of *M*. *edulis* and *M*. *trossulus*. (XLSX)
