# Supplementary figures and images for: Species identification based on a semi-diagnostic marker: Evaluation of a simple conchological test for distinguishing blue mussels Mytilus edulis L. and M. trossulus Gould
Source: PLoS One. 2021 Jul 23;16(7):e0249587. doi: 10.1371/journal.pone.0249587 (PMC8301678; doi:10.1371/journal.pone.0249587)

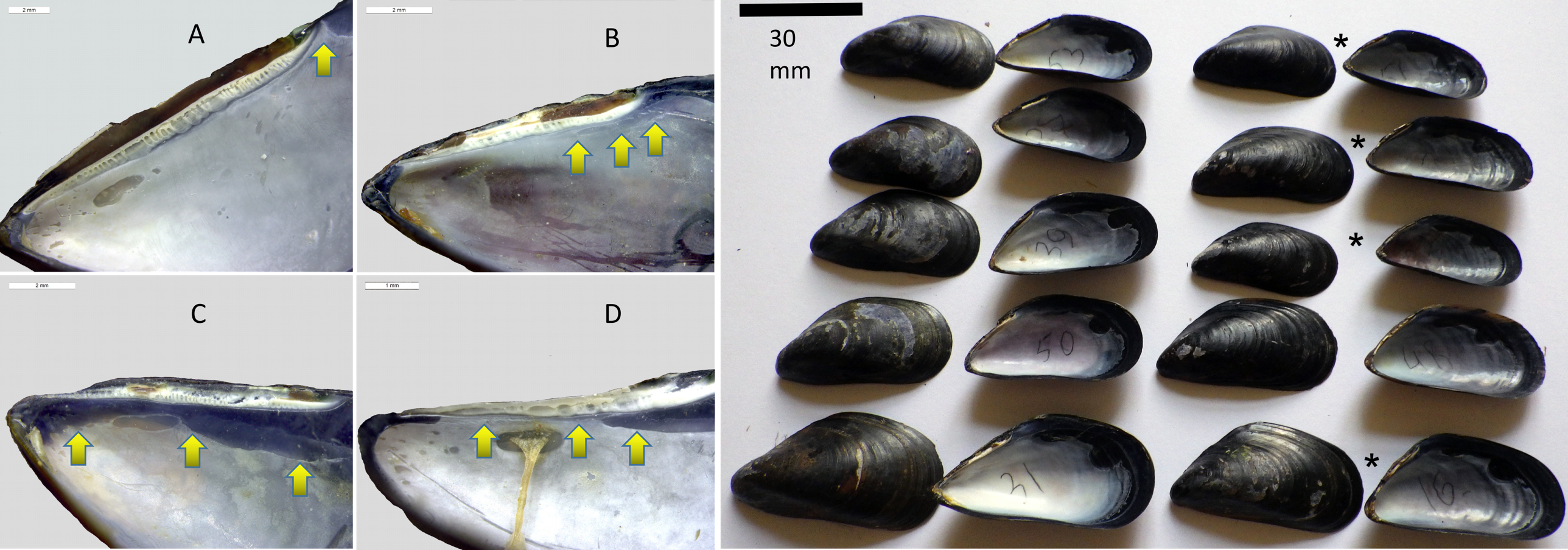

Supplement: S1 Fig — A-D. Stereoscopic micrographs of the ligament area of mussel valves. Note that scale bars differ between A-C and D. Strip of the prismatic layer under the ligament nympha is indicated by arrows. A, B. E-morphotypes: the space under the ligament nympha is totally (A) or partially (B) covered by the nacre. C, D. T-morphotypes: a strip of uncovered prismatic layer under the ligament nympha is dark and wide (C; typical of most examined populations) or pale and narrow, recognizable by a scar separating it from the nacreous layer (D; typical of the Gulf of Maine populations). E. External and internal features of the shell valves of M. trossulus (right) and M. edulis (left) genotypes from the Kola Bay (from sample Sev.17 in S1 Table). In most cases T-morphotypes (marked by *) and E-morphotypes could be distinguished by an unaided eye. (TIFF) [file pone.0249587.s001.tiff]

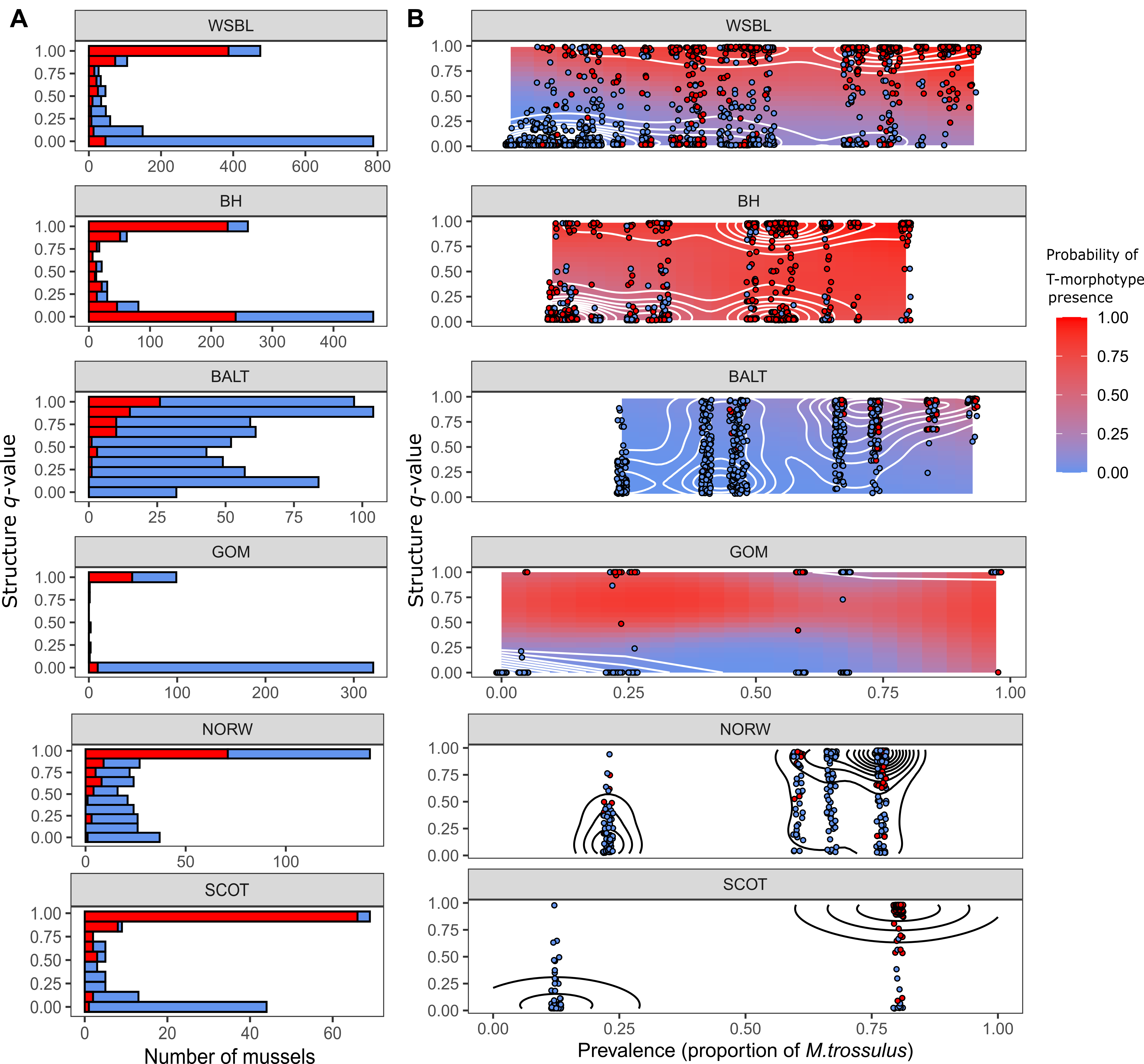

Supplement: S2 Fig — A. Frequency distributions of individual q-values in pooled samples. Red and blue bars indicate T- and E-morphotypes, correspondingly. B. Distributions of individual q-values in samples ordinated by Ptros (proportion of M. trossulus). Red and blue dots indicate T- and E-morphotypes, correspondingly. To avoid overplotting, the horizontal position of all points (individual mussels) was jittered by adding a small random value. For visual purposes, chart areas with maximal dots density are contoured and the probability of T-morphotype presence is shown by the color gradient. The contour lines represent the kernel density estimations (Venables, Ripley 2002) with density2d() function in “ggplot2” package. The probability was assessed using the binomial general additive model, GAM (Zuur, 2012) with the binary outcome (T vs E morphotype) as dependent variable and “Structure q-score”, “Ptros” and “Set” as independent predictors. SCOT and NORW were not included in GAM due to poore cover of Ptros axis. References for S2 Fig: Venables, W. N. and Ripley, B. D. (2002) Modern Applied Statistics with S. Fourth edition. Springer. Zuur, A.F. (2012) A Beginner’s Guide to Generalized Additive Models with R. Highland Statistics Ltd, Newburgh. (TIF) [file pone.0249587.s002.tif]

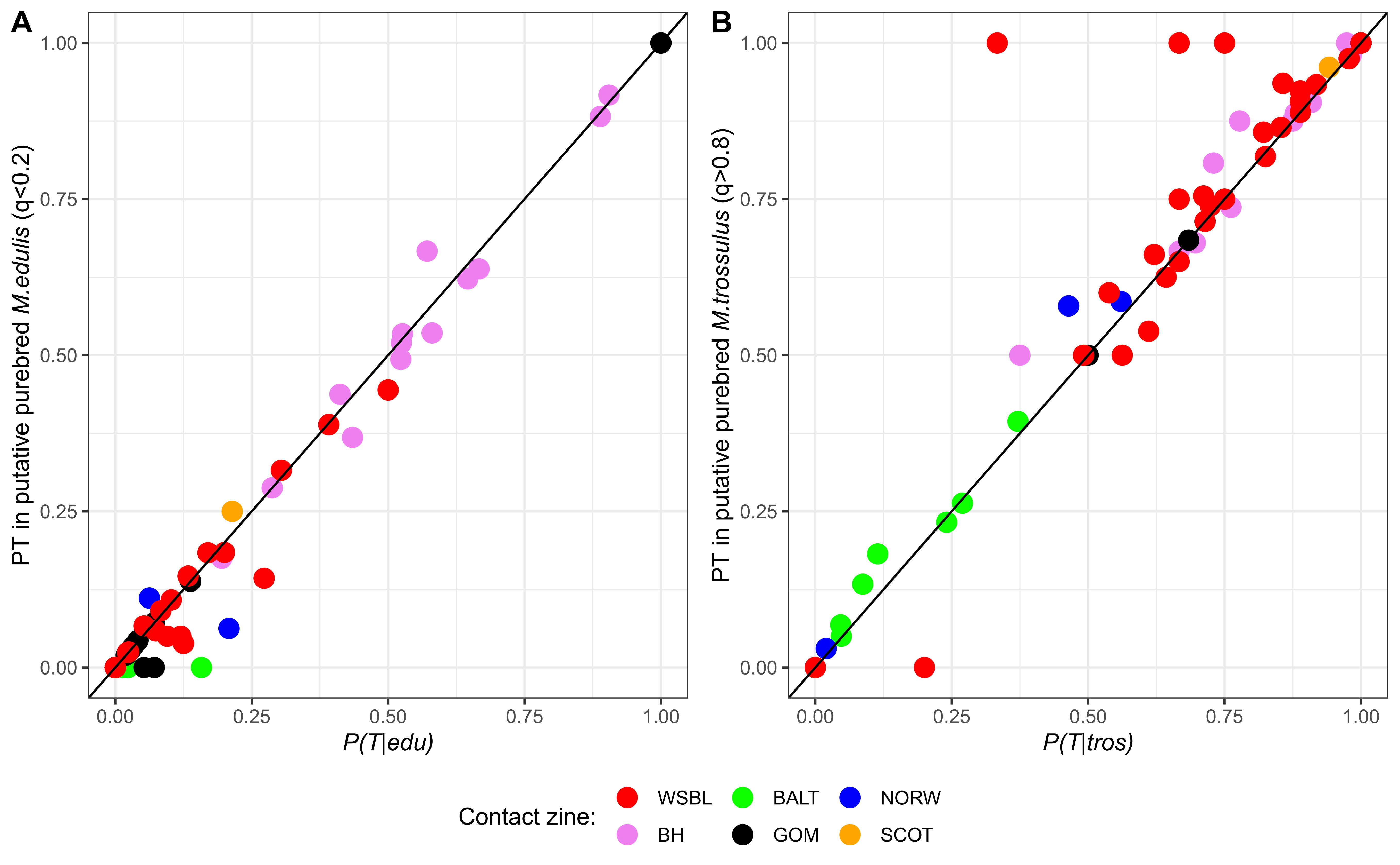

Supplement: S3 Fig — A. M. edulis. B. M. trossulus. Samples from different zones are shown in different colors. (TIF) [file pone.0249587.s003.tif]

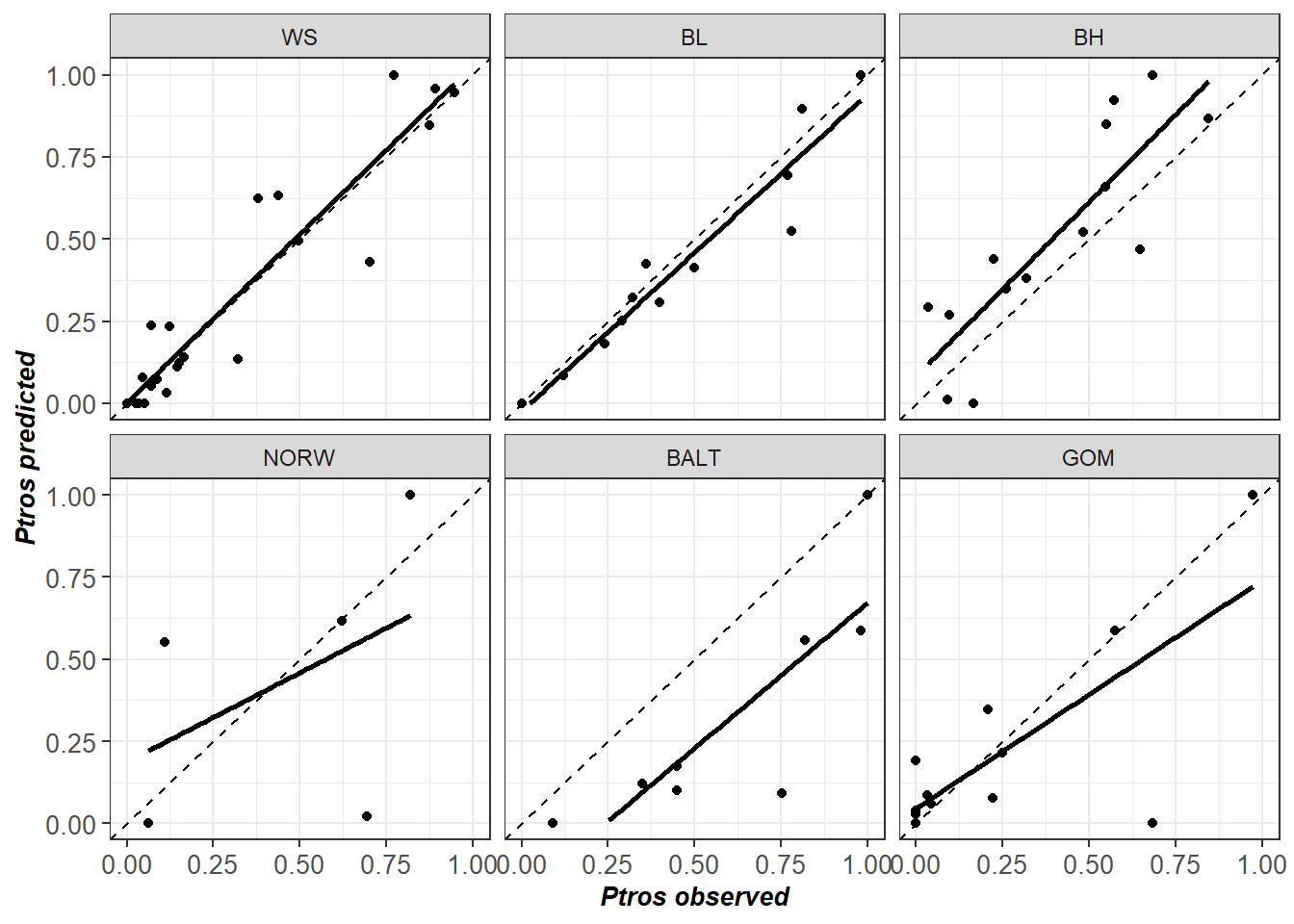

Supplement: S4 Fig — Dots–estimates, solid line–linear regression, dashed line–Y = X line. (TIF) [file pone.0249587.s004.tif]
