# Supplementary material for: Species identification based on a semi-diagnostic marker: Evaluation of a simple conchological test for distinguishing blue mussels Mytilus edulis L. and M. trossulus Gould
Source: PLoS One. 2021 Jul 23;16(7):e0249587. doi: 10.1371/journal.pone.0249587 (PMC8301678; doi:10.1371/journal.pone.0249587)
Supplement: S3 Table — (PDF) [file pone.0249587.s007.pdf]

S3 Table. Parameters of the fitted regression models.

Terms	Estimate	SE	z-statistic	p-value
**Model 1** (GLM)	$pseudoR^2$ = 0.38			
(Intercept)	-2.7	0.15	-18.23	< 0.001
Ptros	4.7	0.28	16.58	< 0.001
$Set_{(BL)}$	0.2	0.26	0.62	0.537
$Set_{(BH)}$	2.2	0.19	11.30	< 0.001
Ptros:$Set_{(BL)}$	-0.5	0.45	-1.03	0.302
Ptros:$Set_{(BH)}$	-1.4	0.43	-3.38	0.001
**Model 2** (GLMM)	$pseudoR^2_m$ = 0.57	$pseudoR^2_c$ = 0.64		
(Intercept)	-4.2	0.38	-10.89	< 0.001
Ptros	4.7	0.82	5.80	< 0.001
$Set_{(BL)}$	0.3	0.75	0.40	0.688
$Set_{(BH)}$	3.5	0.57	6.18	< 0.001
$Species_{(M.trossulus)}$	4.2	0.45	9.34	< 0.001
Ptros:$Set_{(BL)}$	-1.9	1.51	-1.24	0.214
Ptros:$Set_{(BH)}$	-1.8	1.29	-1.36	0.174
Ptros:$Species_{(M.trossulus)}$	-2.5	0.83	-3.02	0.003
$Set_{(BL)}$:$Species_{(M.trossulus)}$	-0.5	0.77	-0.61	0.54
$Set_{(BH)}$:$Species_{(M.trossulus)}$	-3	0.62	-4.87	< 0.001
Ptros:$Set_{(BL)}$:$Species_{(M.trossulus)}$	2.2	1.46	1.52	0.129
Ptros:$Set_{(BH)}$:$Species_{(M.trossulus)}$	2.5	1.25	1.96	0.05
sd(Intercept)	0.8			
**Model 3** (GLMM)	$pseudoR^2_m$ = 0.4	$pseudoR^2_c$ = 0.42		
(Intercept)	3.8	0.28	13.99	< 0.001
$Morph_{(T)}$	-3.8	0.41	-9.12	< 0.001
Ptros	-5.2	0.55	-9.57	< 0.001
$Set_{(BL)}$	-0.4	0.47	-0.88	0.377
$Set_{(BH)}$	-0.6	0.47	-1.21	0.226
$Morph_{(T)}$:Ptros	8.1	0.78	10.40	< 0.001
$Morph_{(T)}$:$Set_{(BL)}$	0.8	0.73	1.09	0.276
$Morph_{(T)}$:$Set_{(BH)}$	-1.6	0.58	-2.71	0.007
Ptros:$Set_{(BL)}$	0.8	0.9	0.91	0.361
Ptros:$Set_{(BH)}$	0.4	1.02	0.36	0.72
$Morph_{(T)}$:Ptros:$Set_{(BL)}$	-0.3	1.37	-0.21	0.83
$Morph_{(T)}$:Ptros:$Set_{(BH)}$	1.4	1.2	1.16	0.244
sd_(Intercept)	0.3			

Terms	Estimate	SE	z-statistic	p-value
**Model 4** (GLM)	$pseudoR^2$ = 0.42			
(Intercept)	-2.4	0.11	-21.34	< 0.001
PT	5.4	0.26	20.74	< 0.001
$Set_{(BH)}$	-1.5	0.32	-4.55	< 0.001
$Set_{(GOM)}$	0.1	0.22	0.69	0.492
$Set_{(BALT)}$	1.8	0.16	11.01	< 0.001
$Set_{(NORW)}$	1.9	0.22	8.91	< 0.001
PT:$Set_{(BH)}$	-0.4	0.5	-0.87	0.386
PT:$Set_{(GOM)}$	0.8	0.74	1.04	0.299
PT:$Set_{(BALT)}$	6.1	1.22	5.05	< 0.001
PT:$Set_{(NORW)}$	-1.8	0.62	-2.81	0.005
**Model 5** (GLMM)	$pseudoR^2_m$ = 0.57	$pseudoR^2_c$ = 0.66		
(Intercept)	-4.2	0.36	-11.64	< 0.001
Ptros	4.2	0.74	5.70	< 0.001
$Set_{(BH)}$	3.6	0.62	5.77	< 0.001
$Set_{(GOM)}$	0.4	0.63	0.55	0.579
$Set_{(BALT)}$	-2.8	1.7	-1.63	0.102
$Set_{(NORW)}$	1.3	1.05	1.27	0.205
$Species_{(M.trossulus)}$	4.1	0.37	11.04	< 0.001
Ptros:$Set_{(BH)}$	-1.1	1.37	-0.82	0.414
Ptros:$Set_{(GOM)}$	-1.7	1.76	-0.98	0.326
Ptros:$Set_{(BALT)}$	1.3	2.56	0.51	0.612
Ptros:$Set_{(NORW)}$	-5.7	2.04	-2.79	0.005
Ptros:$Species_{(M.trossulus)}$	-1.7	0.68	-2.45	0.014
$Set_{(BH)}$:$Species_{(M.trossulus)}$	-2.9	0.57	-5.16	< 0.001
$Set_{(GOM)}$:$Species_{(M.trossulus)}$	0.5	0.98	0.52	0.605
$Set_{(BALT)}$:$Species_{(M.trossulus)}$	-1.4	1.64	-0.85	0.397
$Set_{(NORW)}$:$Species_{(M.trossulus)}$	-2.3	1.28	-1.82	0.069
Ptros:$Set_{(BH)}$:$Species_{(M.trossulus)}$	1.6	1.17	1.41	0.159
Ptros:$Set_{(GOM)}$:$Species_{(M.trossulus)}$	-2.1	2.02	-1.04	0.296
Ptros:$Set_{(BALT)}$:$Species_{(M.trossulus)}$	-0.4	2.41	-0.17	0.863
Ptros:$Set_{(NORW)}$:$Species_{(M.trossulus)}$	3.5	2.03	1.73	0.083
sd(Intercept)	0.9			

Terms	Estimate	SE	z-statistic	p-value
**Model 6** (GLMM)	$pseudoR_m^2$ = 0.5	$pseudoR_c^2$ = 0.51		
(Intercept)	3.7	0.21	17.23	< 0.001
$Morph_{(T)}$	-3.5	0.33	-10.50	< 0.001
Ptros	-4.9	0.41	-12.00	< 0.001
$Set_{(BH)}$	-0.4	0.43	-1.00	0.318
$Set_{(GOM)}$	1	0.58	1.78	0.074
$Set_{(BALT)}$	-0.9	0.41	-2.28	0.023
$Set_{(NORW)}$	-0.6	0.61	-1.00	0.315
$Morph_{(T)}$:Ptros	8.1	0.63	12.90	< 0.001
$Morph_{(T)}$:$Set_{(BH)}$	-1.8	0.53	-3.43	0.001
$Morph_{(T)}$:$Set_{(GOM)}$	-1.8	0.84	-2.18	0.029
$Morph_{(T)}$:$Set_{(BALT)}$	0.4	1.54	0.23	0.82
$Morph_{(T)}$:$Set_{(NORW)}$	-1.1	1.17	-0.95	0.343
Ptros:$Set_{(BH)}$	0.1	0.93	0.09	0.928
Ptros:$Set_{(GOM)}$	-3.2	1.08	-2.92	0.003
Ptros:$Set_{(BALT)}$	-0.5	0.72	-0.72	0.47
Ptros:$Set_{(NORW)}$	0	0.95	-0.05	0.959
$Morph_{(T)}$:Ptros:$Set_{(BH)}$	1.4	1.1	1.27	0.204
$Morph_{(T)}$:Ptros:$Set_{(GOM)}$	4.8	1.88	2.57	0.01
$Morph_{(T)}$:Ptros:$Set_{(BALT)}$	1.2	2.2	0.55	0.579
$Morph_{(T)}$:Ptros:$Set_{(NORW)}$	3.6	1.94	1.86	0.063
sd(Intercept)	0.3			
